# Diet, Microbiome, and Inflammation Predictors of Fecal and Plasma Short-Chain Fatty Acids in Humans

**DOI:** 10.1016/j.tjnut.2024.08.012

**Published:** 2024-08-20

**Authors:** Andrew Oliver, Zeynep Alkan, Charles B Stephensen, John W Newman, Mary E Kable, Danielle G Lemay

**Affiliations:** 1 USDA-Agricultural Research Service, Western Human Nutrition Research Center, Davis, CA, United States; 2 Department of Nutrition, University of California, Davis, Davis, CA, United States; 3 Genome Center, University of California, Davis, CA, United States

**Keywords:** gut microbiome, short-chain fatty acids, machine learning, diet, inflammation

## Abstract

**Background:**

Gut microbes produce short-chain fatty acids (SCFAs), which are associated with broad health benefits. However, it is not fully known how diet and/or the gut microbiome could be modulated to improve SCFA production.

**Objectives:**

The objective of this study was to identify dietary, inflammatory, and/or microbiome predictors of SCFAs in a cohort of healthy adults.

**Methods:**

SCFAs were measured in fecal and plasma samples from 359 healthy adults in the United States Department of Agriculture Nutritional Phenotyping Study. Habitual and recent diet was assessed using a Food Frequency Questionnaire and Automated Self-Administered 24-h Dietary Assesment Tool dietary recalls. Markers of systemic and gut inflammation were measured in fecal and plasma samples. The gut microbiome was assessed using shotgun metagenomics. Using statistics and machine learning, we determined how the abundance and composition of SCFAs varied with measures of diet, inflammation, and the gut microbiome.

**Results:**

We show that fecal pH may be a good proxy for fecal SCFA abundance. A higher Healthy Eating Index for a habitual diet was associated with a compositional increase in fecal butyrate relative to acetate and propionate. SCFAs were associated with markers of subclinical gastrointestinal (GI) inflammation. Fecal SCFA abundance was inversely related to plasma lipopolysaccharide-binding protein. When we analyzed hierarchically organized diet and microbiome data with taxonomy-aware algorithms, we observed that diet and microbiome features were far more predictive of fecal SCFA abundances compared to plasma SCFA abundances. The top diet and microbiome predictors of fecal butyrate included potatoes and the thiamine biosynthesis pathway, respectively.

**Conclusions:**

These results suggest that resistant starch in the form of potatoes and microbially produced thiamine provide a substrate and essential cofactor, respectively, for butyrate synthesis. Thiamine may be a rate-limiting nutrient for butyrate production in adults. Overall, these findings illustrate the complex biology underpinning SCFA production in the gut.

This trial was registered at clinicaltrials.gov as NCT02367287.

## Introduction

Fermentable dietary fiber plays a critical role in feeding the gut microbiome, a complex ecosystem and genetic powerhouse with an outsized role in human health. Dietary fiber resists digestion by the 17 human-encoded glycoside hydrolases [1] and reaches the colon, where its mode of action depends on the type of fiber. Water-insoluble, nonfermentable fibers can add bulk to the stool, whereas fermentable fibers, such as resistant starch, β-glucans, and water-soluble fibers, feed the dense population of colonic bacteria (reviewed in [2]). Some members of the gut microbiota can break down these complex polysaccharides, providing substrates for specialized fermenters to metabolize into end-products like short-chain fatty acids (SCFAs). SCFAs have been broadly implicated in health benefits, including reduced cancer cell proliferation, decreased inflammation, and maintenance of colonocyte health and gut barrier integrity (reviewed in [3]). Therefore, interventions that increase SCFA production could be helpful in preventing chronic diseases.

The most notable SCFAs are acetate, propionate, and butyrate, which can be measured in a fecal sample occurring at a ratio of 60:20:20, respectively [4]. Prior to excretion, roughly 95% of SCFAs produced are either metabolized by resident microbes or absorbed through the colonocytes lining our colon, where their fates diverge substantially [5,6]. Most (up to 90%) of the absorbed butyrate gets metabolized in the colonocyte itself, providing 70% of the energy needed for the cell [7]. Although absorbed propionate can serve as a substrate for gluconeogenesis in the liver, little glucose arises from propionate metabolism in humans [6]. As such, 86% (mean) of propionate is rapidly converted to carbon dioxide [6]. Finally, after conversion to acetyl-coenzyme A, acetate can enter the tricarboxylic acid cycle and is subsequently converted to tricarboxylic acid cycle intermediates [8]. Approximately 64% of colonic-derived acetate, 9% of propionate, and 2% of butyrate ultimately enter systemic circulation [6].

Much of what is understood about diet-microbiome-SCFA relationships comes from animal models. Human intervention studies have yielded mixed results, with fecal SCFAs often unaffected by dietary intervention (reviewed in [9]). This could result from the use of isolated fibers or incomplete characterization of dietary fiber content, as well as the limitation that most SCFAs are absorbed and not observable in fecal samples. To address these knowledge gaps, we analyzed fecal and plasma SCFAs from a cohort of well-phenotyped, healthy United States adults with detailed dietary and microbiome data. Finally, we applied a novel approach in machine learning, taxonomically-informed feature reduction, to identify diet-SCFA and microbiome-SCFA relationships.

## Methods

### Participants

The individuals in this study (*n* = 363) were originally recruited for the USDA Nutritional Phenotyping study [10] (Supplemental Figure 1). The original study was powered to explain 3% of the variation in postprandial inflammation markers in response to a challenge meal [10]. This work investigates a secondary objective of the original study, intends to be exploratory, and is not explicitly powered to explain variation in SCFAs. Briefly, participating individuals were healthy adults aged 18–65 y, with a BMI (in kg/m^2^) between <25–44. Individuals were recruited to evenly fill three age and three BMI bins for both males and females (Supplemental Table 1). Exclusion criteria included hypertension, known chronic disease treated with medication, recent surgery, antibiotic use in the previous four weeks, recent hospitalization, and pregnant or lactating females. More information can be found on clinicaltrials.gov (identifier NCT02367287).

### Dietary assessment

Dietary assessment was conducted using the Block Food Frequency Questionnaire (FFQ, NutritionQuest) and Automated Self-Administered 24-h (ASA24) Dietary Assessment Tool [11]. ASA24 dietary recalls were averaged for at least two (but up to three) recalls, which were collected on different days recalls, which were collected on different days, and data was processed and carefully cleaned [12]. The Healthy Eating Index (HEI) [13] was calculated for both dietary assessments. The representation of consumed foods as a dietary taxonomy [14] and the associated diversity metrics for this cohort have been previously published [15].

### Blood sample collection

A blood draw was conducted in the morning, after a 12-h, water-only fast, as previously described [16]. Prior to the fast, a standardized meal was consumed [10]. Sodium heparin or EDTA was used as an anticoagulant. Plasma was collected after refrigerated (4°C) centrifugation at 1300 x g for 10 minutes. Plasma aliquots were transferred to cryo-store vials and stored at –80°C.

### Stool consistency and collection

The stool was collected using a Ziploc bag, immediately placed on ice, and transported to the Western Human Nutrition Research Center for same-day processing, as previously described [17]. The stool sample was collected at the end of the 7–10 d period during which the dietary recalls were collected. The stool consistency was assessed by a trained technician. The stool was homogenized, flash frozen, and stored at –70°C until DNA extraction, pH measurement [18], and other analyses (Supplemental Table 2).

### Measurement of inflammatory markers

Complete blood counts were performed on whole blood treated with EDTA as an anticoagulant. Counts were performed on three different machines over the course of the 4-y recruitment: Beckman Coulter LH750/780, Beckman Coulter DxH800 automated hematology analyzer, or an Abbott Cell-Dyn 322 analyzer. Plasma LPS-binding protein (LBP) (using heparin-treated plasma samples), fecal calprotectin (CAL), and fecal myeloperoxidase (MPO) were all quantified by ELISA kits as previously described [16]. Fecal neopterin was also measured by ELISA, as described in detail elsewhere [19]. C-reactive protein (CRP) was assessed using the V-PLEX Vascular Injury Panel 1 kit (Meso Scale Discovery) as previously described [16,20].

### DNA extraction and sequencing from fecal samples

DNA from fecal samples was extracted using the ZymoBiomics DNA Miniprep kit (Zymo Research), as previously published [16]. Library preparation and sequencing were performed by the DNA Technologies & Expression Analysis Core Laboratory at the University of California Davis Genome Center. Initially, 290 samples were sequenced, analyzed, and have been described in previous publications [16]. An additional 40 samples were sequenced using identical methods, and these shotgun metagenomes appear for the first time in this publication.

### Quantification of fecal SCFA

Approximately 150 mg (145–155 mg) wet stool aliquot per sample was massed out, and exact stool weights were recorded. Each stool sample was spiked with deuterated SCFA surrogates (10 μL mixture of 100,000 μM acetate-d3 and 10,000 μM propionate-d5 in methanol). SCFAs were extracted into 200 μL of a 1:1 methanol:acetonitrile solution by bead beating with a Geno/Grinder 2010 homogenizer (Cole-Palmer) for 8 minutes at 1200 rpm. The extracts were clarified by centrifugation for 10 minutes at 10,000 × *g* at 4°C, and then passed through a 0.2 μm 96-well filter plate (Agilent) by centrifugation for 1 minute at 1000 × *g*. Twenty-five μL of filtrate was mixed with one volume of internal standard 40 μM cis-10-pentadecenoic acid methyl ester and run on a 7890 gas chromatograph interfaced with a 5977B mass selective detector (Agilent). One μL of sample was injected with a 75:1 split ratio and resolved on a 30 m x 0.25 mm inner diameter x 0.25 μm DB-WAX ultra inert column (Agilent). SCFA concentrations were determined from standard curves with the MassHunter software (Agilent), and final concentrations were calculated by correcting for the percentage recoveries of the deuterated surrogates in each sample.

### Quantification of plasma SCFA

Acetic acid (C2), propionic acid (C3), and butyric acid (C4) were measured in EDTA-treated plasma samples in the Columbia University Medical Center Biomarkers Core Laboratory using ultra-performance liquid chromatography-tandem mass spectrometry. Samples were spiked with deuterated internal standards and subjected to protein precipitation followed by derivatization with 3-nitrophenylhydrazine [21]. The derivatized metabolites were separated on a 2.1 × 100 mm, 1.7 μm ACQUITY BEH C_18_ UPLC column (Waters) maintained at 50°C by gradient elution with water and acetonitrile containing 0.1% formic acid as mobile phases at a flow rate of 400 μL/min. Liquid chromatography-tandem mass spectrometry analysis was performed using positive electrospray ionization with multiple reaction monitoring modes on a Waters Xevo TQS MS - ACQUITY UPLC system (Waters).

### Taxonomic and functional profiling of microbiome

For a subset of 330 individuals, raw metagenomic reads were processed as previously described [16], resulting in merged reads output from FLASH (v.1.2.11) [22]. These reads were used as input to MetaPhlAn (v4.0.6) [23] against the vOct22 database, using default parameters and the --add_viruses flag.

The same reads used in taxonomic profiling were input to HUMAnN (v3.7) [24] using the default parameters and the flag --search-mode uniref90. The ChocoPhlAn database used was v201901_v31, and the uniref90 database was v201901b_full. Pathway abundance files were merged and normalized to copies per million using the humann_renorm_table command.

The merged reads were also used to analyze the inferred fiber degradation profile (IFDP) [25]. IFDP (v1.0.0) was performed with modification. Briefly, the resulting counts from IFDP mapping to fiber degradation enzymes were divided by the protein length and then by the sample-specific genome equivalents, calculated using MicrobeCensus [26]. The resulting counts represent reads per kilobase per genome equivalent.

### Hierarchical feature engineering of microbial taxa and consumed foods

Separately for each SCFA, the taxonomic microbiome profiles and/or reportedly consumed foods (i.e., dietary taxonomy) were feature-engineered using a hierarchical feature engineering program called TaxaHFE (v2.0) [27]. Along with the food features and the microbial features, covariates age, sex, and BMI were also included in the feature engineering. For fecal samples specifically, we also included stool weight and a trichotomous stool consistency factor (hard: 1–2 on the Bristol stool chart; normal: 3–5; and soft: 6–7) [17] as a covariate. TaxaHFE was analyzed with the abundance filter set to 0, the prevalence filter set to 0.01, the lowest level (-L) set to 3, and the number of permutations set to 80. Because of our small sample size, we analyzed TaxaHFE on the entire sample set and fed these engineered features into downstream machine learning. As such, our results (i.e., model scores and features of importance) describe our cohort specifically and are not intended to be broadly generalizable. We also applied TaxaHFE to just the training data prior to machine learning and presented the scores of these models, which may be more generalizable but suffer from even fewer samples.

### Statistical analysis

Statistical analyses were performed in R (v4.2.1). The skewness() function from the package moments (v0.14.1) [28] was used to analyze the distribution of the SCFA. To investigate whether SCFAs varied with independent variables of interest [average fiber (ASA24) consumption over recalls, fiber intake (ASA24) per kcal, fiber intake (FFQ), fiber intake (FFQ) per kcal, soluble fiber intake (FFQ), HEI total score (ASA24), HEI total score (FFQ), phylogenetic diversity of carbohydrate foods (ASA24), and phylogenetic diversity of fiber foods (ASA24)], we fitted a linear model with normalized fecal SCFAs (using the bestNormalize (v1.9.1) [29] R package) as the response and included age, sex, and BMI (and stool weight and Bristol stool score for fecal samples) with the explanatory variables. Because plasma SCFA measurements for butyrate and propionate were left-censored, we used a tobit model from the R package VGAM [30] instead of a linear regression model. The linear model or tobit model was then used in a partial correlation analysis using the avPlots() function from the car package (v3.1–2) [31] and the stats::cor.test() function was used to test the significance of the partial correlations. *P* values were corrected using the false discovery rate method (presented as *p.adjust*) within each SCFA or SCFA-ratio when correlating SCFAs to dietary variables related to our directed hypotheses or to inflammation variables. To correlate fecal and plasma SCFAs, the corrr package (v0.4.4) [32] was used, and the correlation method was set to Pearson. Alpha diversity analysis was performed using the R package picante (v1.8.2) [33], specifically calculating Faith’s phylogenetic diversity using the MetaPhlAn provided phylogenetic tree (mpa_vOct22_CHOCOPhlAnSGB_202212.nwk). Beta diversity was analyzed using the adonis2 function from vegan [34], and an example formula is as follows: community_matrix ∼ BMI + Age + Sex + StoolConsistencyClass + StoolWeight + Butyrate, method = “bray,” permutations = 999, by = “terms.” Plotting was performed using ggPlot2 (v3.4.0) [35] with the additional ggpubr package [36], and in the case of the plotted taxonomic trees, the metacoder package was used [37].

### Machine learning

A machine learning (ML) pipeline, based around the Tidymodels R package (v1.0.0) [38] and a random forest model (https://github.com/aoliver44/nutrition_tools), was used to evaluate the predictive capacity of the different datasets and the abundance of each SCFA. Along with the response variables (i.e., SCFAs) and input features, important covariate features such as age, sex, and BMI were also included. In predicting fecal SCFAs, stool sample weight and stool hardness (soft, normal, and hard) were also included. Initially, a null model was fitted in order to compare a trained model. Briefly, to train the ML models, input data was split into 80% training and 20% testing sets. Within the training set, 10-fold 3 times repeated stratified cross-validation was performed. In every fold, a correlation-based feature engineering step was implemented, involving the removal of features with a Pearson correlation coefficient exceeding 0.95, along with the elimination of features with zero variance. Random forest hyperparameters were tuned during this step using a Bayesian optimization hyperparameter search, which was allowed to analyze 160 parameter combinations or for 20 min, whichever came first. Tuning was preemptively ended if 10 consecutive parameter combinations failed to result in a decrease in the mean absolute error (MAE). The best-scoring model’s parameters were used in a final model fitted to the left-out test data, and model scores were collected. This entire process was repeated across 10 random seeds to account for variability in the initial test-train splits. The test data scores were collected for each random seed and averaged. We chose to present the percent change in MAE over the null model, which shows how well an ML model performs compared to a model that predicts the mean response variable. Feature importance was calculated using SHapley Additive exPlanations (SHAP) values and the fastshap R package [39]. The model used to calculate feature importance was the model that performed the best (lowest MAE score) in the 10 random seeds. To plot SHAP values, we used the shapviz R package [40]. This machine learning pipeline can be found in the above GitHub repository, and a Docker container is provided to aid in reproducibility.

## Results

### Fecal and plasma SCFAs vary across healthy individuals and are negatively correlated with each other

**FIGURE 1:**
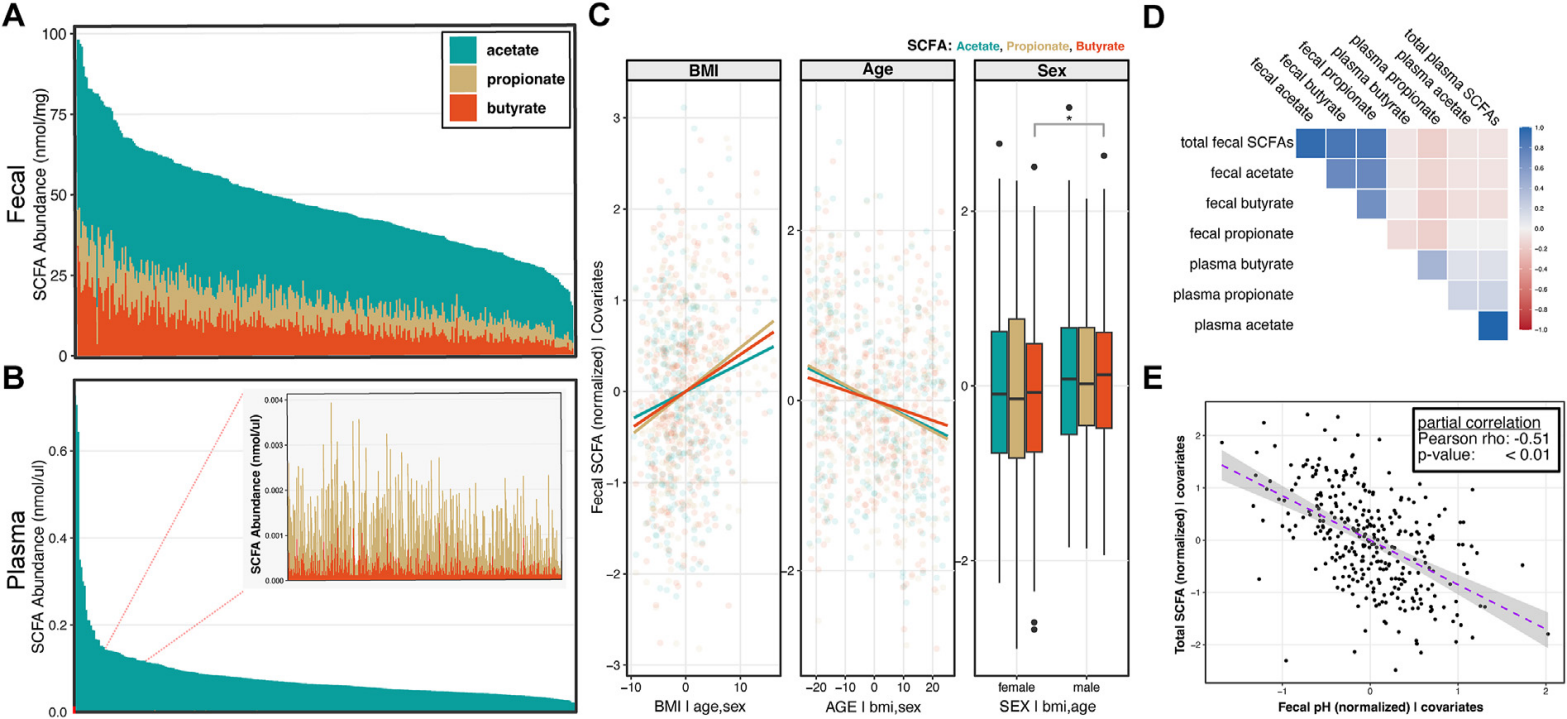
An overview of SCFA abundances in a healthy United States cohort. (A) The abundances of fecal SCFAs (acetate, propionate, and butyrate) for 313 individuals. (B) The abundance of plasma SCFAs (acetate, propionate, and butyrate) for 315 individuals. (C) Partial correlation between each fecal SCFA and either BMI, age, or sex. (D) Pearson correlations between fecal and plasma SCFAs. (E) Partial correlation between fecal pH and total fecal SCFAs, adjusting for age, sex, BMI, stool weight, and stool consistency in the model. BMI, body mass index; SCFA, short-chain fatty acid.

We collected stool samples from 363 individuals; however, fecal samples (*n* = 50) were removed from downstream analysis if they were not returned to the study site within 24 h. We quantified fecal SCFAs (acetate, propionate, and butyrate) for 313 individuals and plasma SCFAs (acetate, propionate, and butyrate) for 315 individuals (Figure 1A and B**)**. Altogether, we measured fecal and/or plasma SCFAs for 359 individuals, of which 269 individuals had both measurements. For fecal samples, acetate was the most abundant SCFA measured (mean = 29.56 nmol/mg, SD = 8.73), followed by butyrate (9.49 ± 5.31) and propionate (8.56 ± 3.57). The abundances of fecal SCFAs were all positively skewed across individuals. Across all subjects, the proportions of acetate:propionate:butyrate occurred at 61:17:18, approaching the often-reported ratio of 60:20:20. Fecal SCFA abundances (acetate, propionate, and butyrate) changed slightly but significantly (partial correlation, *P* < 0.01) with anthropometrics: increasing with BMI and decreasing with age (Figure 1C). Only fecal butyrate was significantly higher in males than in females (partial correlation: *P* < 0.05).

For plasma samples, nearly the entire fraction (98%) measured was acetate. On average, acetate accounted for 0.08 nmol/μL ± 0.08 of the plasma SCFA abundances, followed by propionate (9.5e^–4^ nmol/μL ± 5.9e^–4^) and butyrate (2.8e^–4^ nmol/μL ± 2.0e^–4^). In contrast to fecal SCFAs, plasma SCFAs were not impacted by differences in anthropometrics, such as age, sex, and BMI (partial correlation: *P* > 0.05).

Fecal SCFAs were positively correlated among themselves, as were plasma SCFAs (Figure 1D). The total abundance of fecal SCFAs was weakly and negatively correlated with the total abundance of plasma SCFAs (*r* = –0.0685, *P* = 0.263). For a single SCFA, this inverse relationship was most apparent for propionate, where increases in fecal propionate were associated with decreases in plasma propionate (*r* = –0.168, *P* = 0.006). The weakest correlation between a fecal SCFA and its plasma counterpart was butyrate (Pearson’s rho = –0.0279, *P* = 0.649).

### Fecal pH is a good proxy for fecal SCFA abundance but not plasma SCFAs

We next investigated whether fecal pH was correlated with SCFA abundances. Unsurprisingly, as fecal SCFAs increased, fecal pH decreased. Total fecal SCFAs were significantly correlated with fecal pH (partial correlation: *r* = –0.51, *P* < 0.001) (Figure 1E). Fecal butyrate had the strongest association, with a partial correlation of –0.53 (*P* < 0.001), followed by acetate (*r* = –0.48, *P* < 0.001) and propionate (*r* = –0.33, *P* < 0.001). In contrast, none of the plasma SCFAs were correlated with fecal pH.

### SCFA composition is associated with habitual healthy eating

We assessed whether there were aspects of diet that influenced the abundance and composition of fecal and plasma SCFAs. First, we tested our hypothesis that SCFAs would positively correlate with healthy dietary patterns, measured using the HEI. After correcting for multiple comparisons, neither raw fecal nor raw plasma SCFAs significantly correlated with the HEI calculated using habitual (FFQ) or recent (ASA24) dietary data. We next asked if these results were influenced by inflammation, which can dysregulate SCFA metabolism in the colon. Again, we found no relationship between fecal or plasma SCFAs and the HEI after removing individuals with frank inflammation (a CAL value >100 μg/g and a plasma CRP value >10 mg/L) (Supplemental Table 2). Because the composition of fecal SCFAs has been shown to occur at a ratio of 60:20:20, we hypothesized that deviations from this expected compositional ratio might correlate with dietary variables better than raw abundances. Indeed, the deviation of fecal butyrate from this ratio (e.g., a positive value indicating relatively more butyrate than expected), referred to as butyrate-ratio henceforth, was significantly correlated with the HEI index calculated using habitual diet (FFQ) (*r* = 0.183, *P.adjust* = 0.013) (Supplemental Figure 2). The relationship between butyrate-ratio and HEI (FFQ) was nearly identical for the subset of samples without frank inflammation (*r* = 0.178, *P.adjust* = 0.064) (Supplemental Figure 2 and Supplemental Table 2).

Our second hypothesis was that SCFA abundance would significantly correlate with dietary fiber intake and diversity. No fecal or plasma SCFAs significantly varied with dietary fiber variables after correcting for multiple comparisons. However, there were notable trends within the entire cohort: butyrate-ratio was positively correlated with calorie-corrected dietary fiber intake (FFQ) (*r* = 0.13, *P* < 0.05, *P.adjust* > 0.05) and the HEI (ASA24) (*r* = 0.13, *P* < 0.05, *P.adjust* > 0.05) (Supplemental Figure 2). Although we expected to find positive relationships between fiber variables and SCFAs (i.e., increased fiber consumption would lead to increased SCFAs), we also found negative trends between soluble fiber intake and plasma propionate (*r* = –0.12, *P* < 0.05, *P.adjust* > 0.05) (Supplemental Figure 2).

### Dietary butyrate is not a large source of measured butyrate in stool or plasma

Because the ASA24 quantifies butyric acid in food (but not acetate or propionate), we asked whether dietary butyrate significantly correlated with the butyrate we measured in plasma and fecal samples. For this analysis, we disaggregated the ASA24 recalls to the individual days they were recorded and chose subjects who supplied a food recall that was within 0–2 d before a stool sample was collected (*n* = 95 individuals). However, we did not detect any relationships between fecal or plasma butyrate and dietary butyrate (Supplemental Figure 3).

### Fecal SCFAs inversely associate with markers of inflammation; plasma propionate inversely associates with GI inflammation

Next, we examined whether SCFAs varied with gut or systemic inflammation. Individuals with plasma CRP ≥10 mg/L were considered high-inflammation individuals (32 fecal samples and 34 plasma samples), compared to normal-CRP individuals (273 fecal samples and 279 plasma samples) (Supplemental Figure 4A). We found no significant differences in the abundance of any SCFA, plasma or fecal, comparing high to normal inflammation individuals (Wilcoxon, *P* > 0.05) (Supplemental Figure 4B). We also examined gut inflammation, using a CAL cutoff ≥100 μg/g to designate high-inflammation individuals (45 fecal and plasma samples) and normal individuals (268 fecal samples and 265 plasma samples). Similarly, we found no differences in the abundances of SCFAs between high and normal-CAL individuals (Supplemental Figure 4C).

Because not all markers of inflammation have established high-inflammation thresholds, we also assessed inflammation with partial correlation. We have previously found more pronounced relationships between diet and the abundance of inflammatory markers when individuals with frank inflammation are removed [41]. Therefore, we removed individuals with high CAL and/or high plasma CRP and assessed the relationship of various inflammatory markers with SCFA abundance in the presence of covariates (age, sex, BMI, and fecal samples - stool consistency and stool weight). Our partial correlation analysis revealed several significant relationships between SCFA and markers of inflammation (Table 1). Specifically, plasma propionate was negatively associated with MPO (partial correlation *r* = –0.259, *P.adjust* = 0.0002, Supplemental Figure 5A). Fecal SCFAs were also correlated with several markers of inflammation. Notably, acetate, propionate, and butyrate were all negatively correlated with plasma LBP (Table 1, Supplemental Figure 5B–E).

**TABLE 1:** Table showing the significant partial correlations between fecal and plasma SCFAs and various markers of inflammation.

Type	SCFA	Factor	Correlation estimate	Correlation P value	Regression P value	N individuals	Tobit estimate	Tobit P value
Fecal	Acetate-ratio	Fecal MPO	–0.146	0.025	0.027	235	–	–
Fecal	Acetate-ratio	Fecal neopterin	0.157	0.017	0.018	230	–	–
Fecal	Acetate	Plasma LBP	–0.151	0.021	0.022	235	–	–
Fecal	Propionate	Plasma LBP	–0.132	0.043	0.045	235	–	–
Fecal	Butyrate	Plasma LBP	–0.143	0.028	0.03	235	–	
Fecal	Total SCFA	Plasma LBP	–0.161	0.013	0.014	235	–	–
Fecal	Propionate	White blood cell count	0.146	0.026	0.028	233	–	–
Fecal	Butyrate	White blood cell count	0.14	0.033	0.035	233	–	–
Fecal	Total SCFA	White blood cell count	0.139	0.034	0.036	233	–	–
Plasma	Propionate	Fecal MPO	–0.26	0	–	235	–0.001	0
Plasma	Butyrate	Fecal neopterin	–0.135	0.041	–	230	–0.008	0.036

For plasma butyrate and propionate, tobit models were used for censored regression. Covariates for plasma SCFA models were age, sex, and BMI. For fecal SCFA models, covariates were age, sex, BMI, stool weight, and stool consistency (Bristol stool score). Regression *P* -values were family-wise adjusted within each SCFA, using the false discovery method.

Abbreviations: BMI, body mass index; LBP, lipopolysaccharide binding protein; MPO, fecal myeloperoxidase; SCFA, short-chain fatty acid.

### SCFAs associated with differences in gut microbiome diversity and composition

To test whether measures of alpha diversity correlate with SCFA abundance, we performed partial correlations between Faith’s phylogenetic diversity and the abundance of SCFAs. The phylogenetic diversity of the microbiome positively correlated with fecal acetate-ratio (*r* = 0.30, *P* < 0.001) and negatively correlated with butyrate-ratio (*r* = –0.24, *P* < 0.001) and propionate-ratio (*r* = –0.28, *P* < 0.001) (Supplemental Figure 6A). The raw values of butyrate (*r* = –0.21, *P* = 0.002) and propionate (*r* = –0.22, *P* < 0.001) were also negatively correlated with phylogenetic diversity, but acetate (*r* = –0.05, *P* > 0.05) was not. We found no significant correlations between the phylogenetic diversity of the microbiome and plasma SCFA abundances.

We also tested whether SCFA abundance could explain significant variability in microbiome community composition. We used a permutational multivariate analysis of variance (PERMANOVA) model to analyze community composition at every taxonomic level, in addition to TaxaHFE-engineered microbiome features. Surprisingly, for most fecal SCFAs (both raw abundances and ratios), summarizing community composition at the kingdom level explained significant variation (mean *R*^*2*^ = 0.043, Supplemental Figure 6B). Similar to our alpha diversity findings, plasma SCFAs poorly explained compositional differences in the microbiome. On average, TaxaHFE-engineered features best captured compositional variability with respect to plasma SCFA abundance, explaining an average 1.4% variation in the PERMANOVA models (Supplemental Figure 6B). The most variation explained in the microbiome came from relating fecal propionate-ratio to TaxaHFE-engineered microbiome features (*R*^*2*^ = 0.101, Supplemental Figure 6B). The taxa with the largest positive coefficients in the fecal propionate-ratio model included the phylum *Bacteroidetes* and the class *Negativicutes* (Supplemental Figure 7A).

### Machine learning models identify dietary and microbiome features predictive of SCFA abundance

**FIGURE 2:**
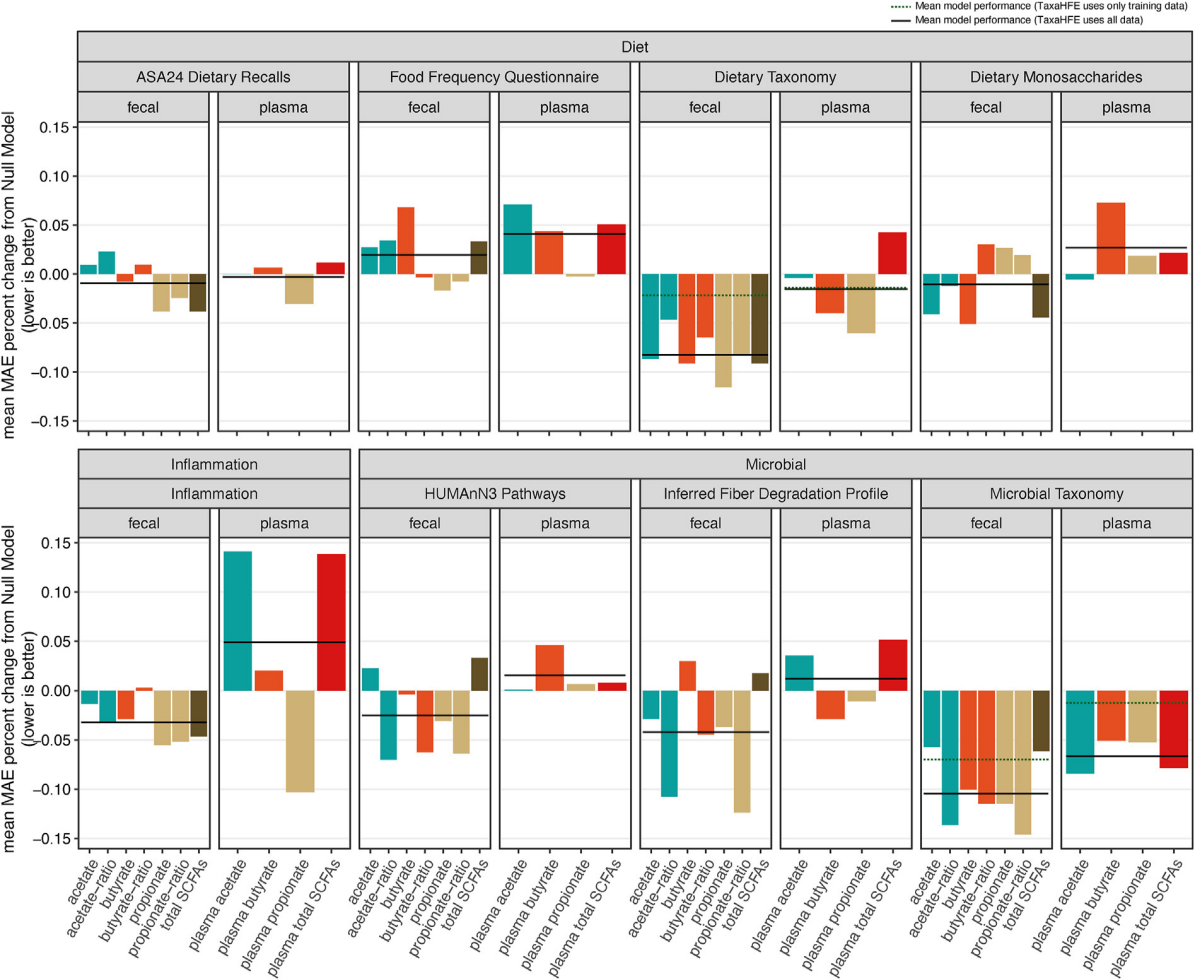
Predicting SCFA abundances using machine learning. The mean MAE percent change between a trained machine model and a null model for fecal and plasma SCFAs using dietary, inflammation, and microbiome features. Solid horizontal lines represent the mean MAE percent change over the null model for all SCFAs of a given data type. When TaxaHFE was employed, solid lines indicate that TaxaHFE was trained on all samples prior to machine learning, and dashed lines indicate TaxaHFE was trained on only the training sample subset. ASA24, Automated Self-Administered 24-h Dietary Assessment Tool; MAE, mean absolute error; SCFA, short-chain fatty acid; TaxaHFE, Taxonomic Hierarchical Feature Engineering; HUMAnN3, HMP Unified Metabolic Analysis Network.

Outside of our directed hypotheses, we implemented machine learning to investigate which components of diet, the microbiome, or inflammation markers are associated with individual SCFAs. We tested eight different types of predictor variables and their ability to lower MAE relative to a null model (Figure 2; Supplemental Table 3). These eight data types were as follows: *1*) ASA24 recent diet, *2*) FFQ habitual diet, *3*) TaxaHFE-engineered recent diet, *4*) dietary monosaccharides, *5*) TaxaHFE-engineered microbial taxa, *6*) HUMAnN pathways, *7*) IFDP, and *8*) inflammation and immune markers. We permuted each ML analysis over 10 different random seeds. The mean coefficient of variation of our chosen scorer (MAE) for trained models was 8.6% (SD = 3.9%) and 2.3% (SD = 1.8%) for null models.

Although we expected to see dietary features from the FFQ and ASA24 predict SCFAs, features from these datasets provided little to no increase in information over the null models. Interestingly, dietary taxonomy data (foods from ASA24, arranged in a hierarchy), processed with TaxaHFE, was the most predictive dietary feature set for both fecal and plasma SCFAs. These consumed foods were better at predicting raw fecal SCFAs (mean MAE percent decrease over a null model = 9.6%) compared to fecal ratio SCFAs (6.5%). Like models built using FFQ and ASA24 features, using dietary monosaccharides features rarely produced models that were more informative than the null model.

**FIGURE 3:**
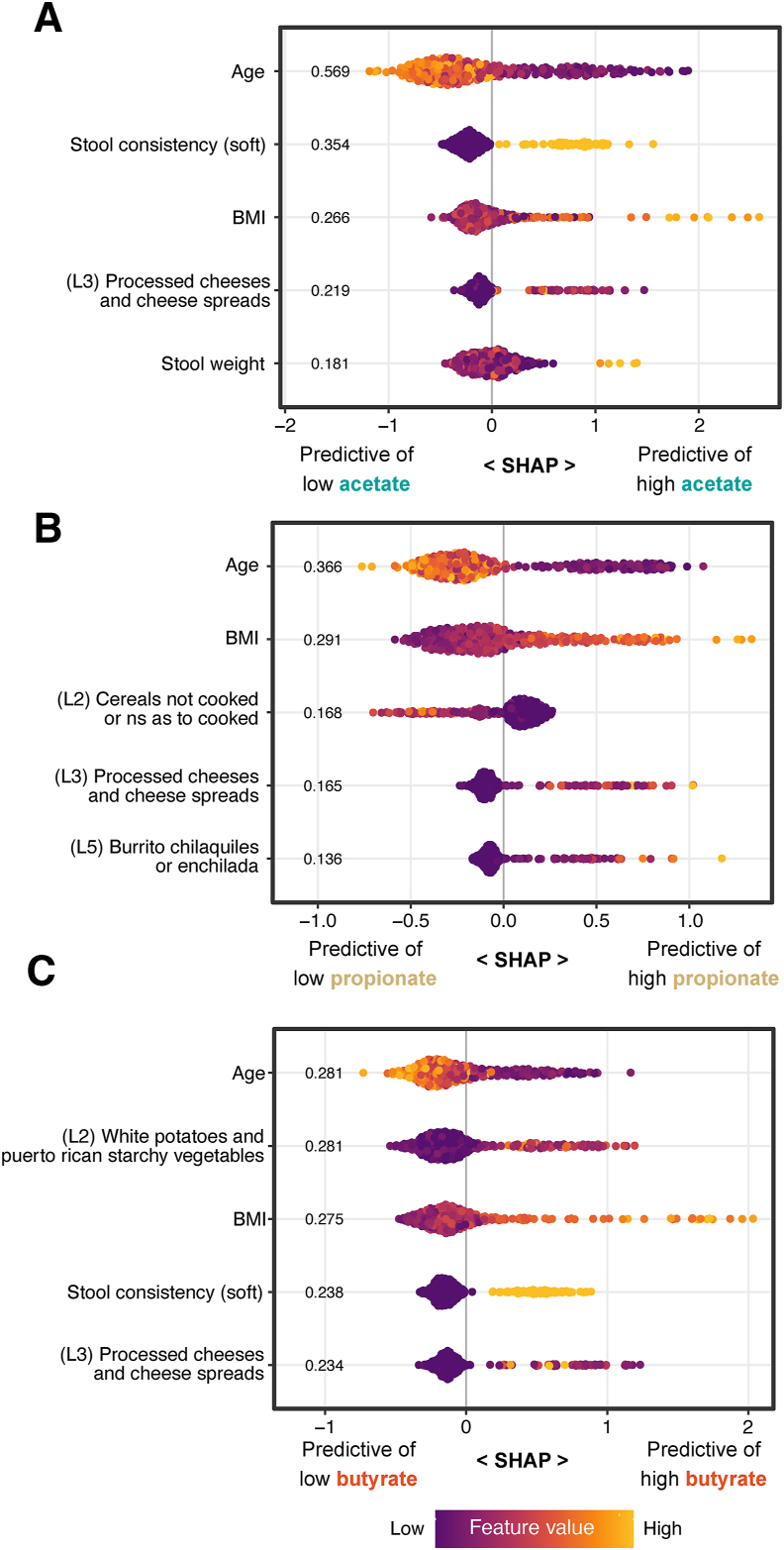
Top features for ML models predicting SCFA abundances from diet. SHAP beeswarm plots showing the top features (by mean absolute SHAP value) for fecal (A) acetate, (B) propionate, and (C) butyrate. BMI, body mass index; SCFA, short-chain fatty acids; SHAP, SHapley Additive explanations; ML, machine learning.

**FIGURE 4:**
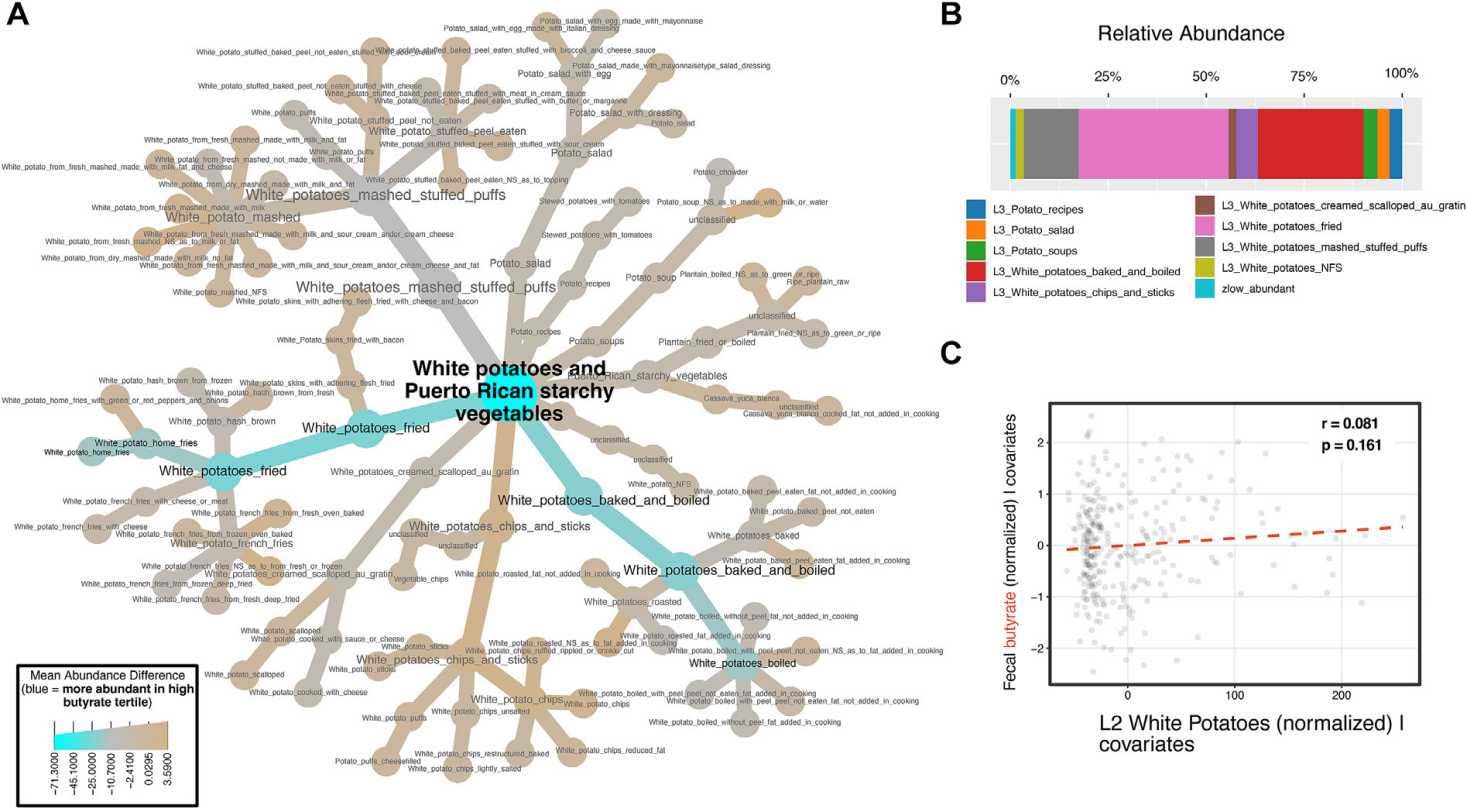
Investigating foods predictive of fecal butyrate. (A) A metacoder taxonomic plot illustrating the food taxa under L2 white potatoes and Puerto Rican starchy vegetables. The colors indicate mean abundance differences in consumption between individuals in the top and bottom tertile for the butyrate-ratio. (B) A stacked bar plot of the most abundant L3 children within the L2 white potatoes and Puerto Rican starchy vegetables. (C) A partial correlation between L2 white potatoes and Puerto Rican starchy vegetables with fecal butyrate.

As the dietary taxonomy models were the most predictive of SCFAs among the dietary models, features contributing to these models were investigated further. In general, person-specific factors such as age, BMI, and stool consistency were stronger predictors than food taxa, but a few foods had predictive values of similar orders of magnitude (Figure 3). The most predictive food taxon of fecal acetate was “Level 3 Processed cheeses and cheese spreads” (Figure 3A; Supplemental Table 4). This taxon was also predictive of fecal butyrate and propionate, with higher processed cheese consumption associated with higher of all three SCFAs. Partial regressions show that the relationship of processed cheese with propionate is the strongest of the three (Supplemental Figure 8). The most predictive food taxon of fecal propionate was “Level 2 Cereals not cooked or not specified as cooked,” being inversely associated with fecal propionate (Figure 3B). This Level 2 Cereals node contains two children, “L3 Ready to Eat Cereals” and “L3 Cereal grains not cooked.” Drilling down further, the most common reports were various types of granola (uncooked oats) and oat-based cereal (also uncooked). Therefore, uncooked cereals, especially oats, appear to be associated with decreased fecal propionate. The most predictive food taxon of fecal butyrate was “L2 White Potatoes and Puerto Rican Starchy Vegetables” (FIGURE 3, FIGURE 4A), which was almost entirely due to the consumption of white potatoes (Figure 4B). A partial regression suggests that white potato consumption may be weakly positively correlated with fecal butyrate (Figure 4C).

We next investigated whether models based on various measures of inflammation and immunity would predict SCFA abundance. These measures of inflammation included CAL, MPO, fecal neopterin, plasma LBP, CRP, and white blood cell count. Inflammation markers were slightly predictive of raw fecal SCFAs (mean MAE percent decrease over null model = 3.6%), and white blood cell count was the most informative feature for these models (after covariates such as age, sex, and BMI). For plasma SCFAs these markers of inflammation were uniquely apt at predicting plasma propionate, with a mean decrease in MAE of 10.3% over the null model. The top feature used in this model was MPO (Supplemental Table 4), supporting our regression model results (Supplemental Figure 5A).

By far, the most useful features of machine learning models were rooted in some aspect of the microbiome. Metabolic pathways predicted using HUMAnN3 were equally predictive of acetate-ratio, propionate-ratio, and butyrate-ratio (mean MAE percent decrease over a null model for all three fecal SCFAs = 6.6%) but not predictive of plasma SCFAs. More performant than HUMAnN3, on average, were models employing features from the IFDP, which similarly produced better models for fecal acetate-ratio, propionate-ratio, and butyrate-ratio than for absolute acetate, propionate, and butyrate. The top features driving the IFDP models included levan degradation capacity for propionate-ratio and rhamnogalacturonan degradation capacity for butyrate-ratio (Supplemental Table 4). Finally, the best-performing models for predicting SCFAs utilized microbial taxonomy as a feature. In contrast to the TaxaHFE-engineered dietary taxonomy, which found raw SCFAs easier to predict, TaxaHFE-engineered microbial taxonomy was uniformly more predictive of SCFA ratios (Figure 2, mean MAE percent decrease over null model for all 3 fecal SCFAs = 13.2%). Unlike any other type of data, microbial taxonomy produced models with lower MAE scores than null models for every SCFA measured (both fecal and plasma).

In general, fecal SCFAs were easier to predict than plasma SCFAs. Of the individual SCFAs, propionate was generally the easiest to predict, regardless of the features used. Moreover, the top-performing model predicted fecal propionate-ratio using TaxaHFE-engineered microbial taxa, which achieved a 14.6% mean MAE percent decrease over the null model. Notably, among the top features driving this model were the phylum *Bacteroidetes* and the class *Negativicutes*, the same taxa we found in the PERMANOVA model (Supplemental Figure 7B).

**FIGURE 5:**
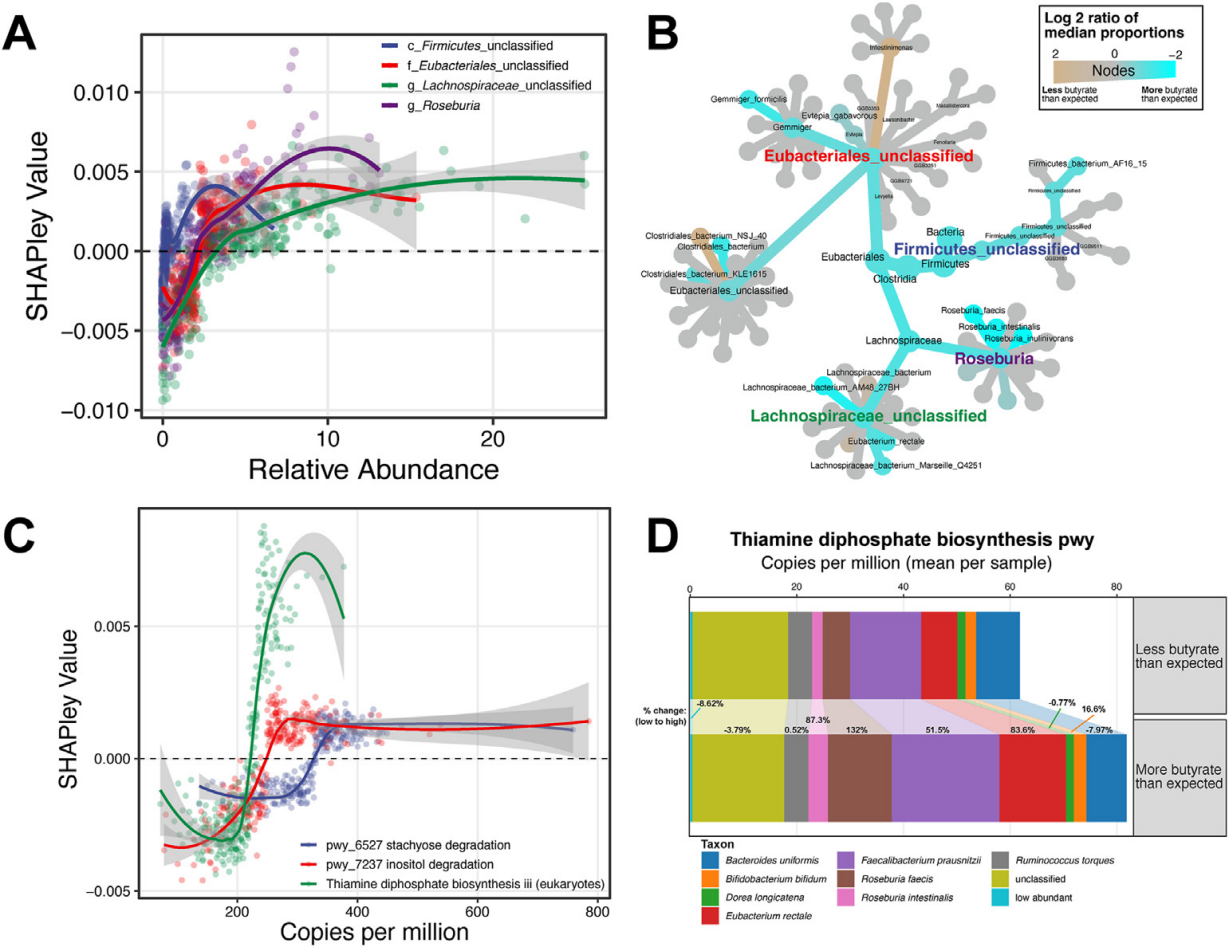
Microbiome-rooted features are predictive of fecal butyrate. (A) Top directly varying (a positive correlation between feature value and SHAP value) TaxaHFE features from microbial taxonomy. These features increase in relative abundance with increasing butyrate-ratio. (B) Finer taxonomic details of the taxonomic groups are presented in (A). The color represents median log-fold differences in abundance between the top and bottom tertiles of butyrate-ratio (C) Top directly varying (a positive correlation between feature value and SHAP value) HUMANnN3 features with butyrate-ratio. (D) The top 9 species of bacteria containing the thiamine diphosphate biosynthesis pathway by mean abundance. SHAP, SHapley Additive explanations; HUMANnN3, HMP Unified Metabolic Analysis Network; TaxaHFE, Taxonomic Hierarchical Feature Engineering.

Because of its recognized role in gut and systemic health, we further investigated the features that drove the top models predicting butyrate. The best model utilized microbial taxonomy to predict butyrate-ratio (mean MAE percent decrease over null model = 10.1%). The top features in these models included known butyrate producers, such as members of the genus *Roseburia* and *Lachnospiraceae* (unclassified) (Figure 5A). When we look further into the species within these genera that may contribute to this signal, we found species such as *Eubacterium rectale* and *Roseburia faecis* differentially abundant between individuals in the highest and lowest butyrate tertiles (Figure 5B). Individuals in our study also differed in the functional potential of their microbial communities. We found that individuals with a higher fecal butyrate-ratio had more reads mapping to stachyose and inositol degradation pathways and to the thiamine diphosphate biosynthesis pathway iii (eukaryotes) (Figure 5C). Of these three pathways, the thiamine diphosphate biosynthesis pathway had the highest mean absolute SHAP value. The thiamine diphosphate biosynthesis pathway is found in a variety of different taxa; however, between high and low butyrate tertiles, we found the most differentially abundant taxa containing this pathway were *E. rectale*, *R. faecis*, and *Roseburia inulinivorans* (Figure 5D). Taken together, these results demonstrate that variations in taxonomic structure are linked to alterations in microbiome functionality, which, in turn, are associated with a higher fecal butyrate composition.

## Discussion

High-resolution diet and microbial data, both organized in taxonomies and analyzed using taxonomy-aware algorithms, provide a new method to understand nutrition and gut microbiomes in free-living humans. Using this approach, we analyzed diet and shotgun metagenomes together with fecal and plasma SCFAs, as well as markers of inflammation in a cohort of over 300 healthy U.S. adults.

First, we found an inverse relationship between total fecal SCFAs and fecal pH. Baxter et al. [42] speculated that lactic acid (pKa of 3.86) produced through the bifid shunt could be responsible, in part, for a decrease in fecal pH. We investigated adding in *Bifidobacterium* abundance as an additional covariate to a model relating fecal pH and total fecal SCFA abundance and found *Bifidobacterium* abundance did not meaningfully increase our ability to explain total fecal SCFAs (Supplemental Figure 9). Although *Bifidobacterium-*produced lactic acid likely still plays a role in lowering colonic pH, we hypothesize that the larger amounts of SCFAs produced [43] likely have a greater impact on fecal pH, even after accounting for the higher pKa of SCFAs compared to lactic acid. Further, we found inverse relationships between fecal SCFAs and plasma SCFAs. As both fecal and plasma SCFAs are difficult to measure and fecal pH quite easy, the relationships demonstrated in the current study suggest that fecal pH could be a cheap and accessible clinical proxy.

Our results show that fecal SCFAs are impacted by age and BMI, and in the case of butyrate, sex (Figure 1D). Salazar et al. [44,45] showed significant decreases in fecal SCFA abundance between middle age (57–67 y) and older individuals (77–95 y). We extend this significant association to younger adults, as the individuals in our cohort aged 18 to 66 y. One review speculated that a decline in the metabolic output of resident gut microbes results in a decrease in SCFA abundance during aging [46] because the abundance of butyrate-producing bacteria is not reduced during aging [44]. Far less is known about the relationship between plasma SCFAs and age. Contrary to our expectations, we found no change in plasma SCFA abundance with age.

One meta-analysis showed obese individuals had higher measured fecal acetate, propionate, and butyrate [47]. We also found a positive correlation between BMI and fecal SCFAs. It has been suggested that SCFAs could contribute 80–200 kcal/d in additional energy for the host [48]. On the surface, this is not a large amount; however, a small net increase in energy intake compounded over time can lead to increases in BMI [49,50]. In contrast, we found no relationship between plasma SCFAs and BMI.

Although we are not the first to examine fecal SCFAs as a ratio or in terms of relative abundances [[51], [52], [53]], our machine learning results (Figure 2) point to an interesting difference between raw and relative abundances of fecal SCFAs: dietary variables appeared to better predict raw fecal SCFAs whereas microbiome variables better predicted fecal SCFA ratios. We initially suspected one reason for this could be technical: dietary variables were used as raw input and not scaled, as opposed to microbiome data, which was total sum scaled. To test this, we analyzed the same dietary taxonomy dataset through the same TaxaHFE + machine learning pipeline, but the raw dietary taxonomy counts were total sum scaled. Although the differences were less apparent, the dietary taxonomy still predicted raw fecal SCFAs better than SCFA relative abundances (Supplemental Figure 10). Thus, a biological reason may explain these results: total carbon sources available determine the total amount of SCFAs produced and the different types of microbes negotiate which kinds of SCFAs are produced.

We found that person-specific factors, such as age, sex, or BMI, more strongly predicted SCFA concentrations than whole diets or specific foods. This is a common theme in nutrition research and is the reason for new efforts in precision nutrition. For example, person-specific characteristics were found to be more predictive of a person’s blood glucose concentrations following a meal than either the carbohydrate or caloric content of that meal [54]. In the current study, when controlling for known person-specific characteristics such as age, sex, and BMI, a habitual healthy eating pattern was associated with a shift toward more fecal butyrate.

Dietary taxonomy was a much better predictor of SCFAs than diet as traditionally aggregated into food groups or nutrients. Traditional dietary analyses do not adequately describe the carbon sources that are available to gut microbes. Given that food groups from a dietary taxonomy were better associated with gut microbes than nutrients [14,15], it is rational that dietary taxonomy would also be a better predictor of microbial metabolites. Information about the carbon sources in foods is beginning to become available, such as the Davis Food Glycopedia [55]. Although a previous study demonstrated an association of specific dietary monosaccharides with specific gut bacteria in the present cohort [19], dietary monosaccharides were not predictive of fecal SCFAs. Additional structural information for the glycochemistry of food is much needed.

Surprisingly, “processed cheeses” were a common predictor of fecal SCFA abundance. This may be because processed cheeses, unlike aged cheeses, contain substantial lactose, and the cheese matrix slows its digestion, perhaps enabling lactose to reach the colon. Moreover, this multiethnic cohort included people who were genetically lactose intolerant [56]. Of these lactose-intolerant individuals, those who consumed higher amounts of lactose exhibited an increase in acetate-producing bacteria [57]. Thus, it’s possible that processed cheese is associated with increased SCFAs due to its lactose content, matrix effects, host genetics, and habitual consumption.

That uncooked cereals, especially uncooked oats, were associated with lower fecal propionate could be because such cereals are low in soluble, fermentable fiber. Cooked oats are high in soluble fiber, but uncooked oats may be less fermentable. There is a dearth of literature on this subject. A randomized crossover dietary intervention trial in 32 adults with 6-wk intervention periods compared 45 g of whole-grain oat granola with nonwhole-grain breakfast cereals, and there was no difference in fecal SCFA concentrations [58]. An intervention study comparing cooked and uncooked oats is much needed.

The dietary taxonomy analysis revealed white potatoes as potentially associated with fecal butyrate. Previous studies have shown that individuals who had resistant starch-degrading bacteria *Ruminococcus* and *Eubacterium* or *Bifidobacterium* present in their gut microbiota produced more fecal butyrate in response to a potato-resistant starch intervention than those who did not [42,59]. Moreover, butyrate production from potato-derived resistant starch tended to be higher than from other fiber sources. In a prior analysis of this cohort [15], gut microbial communities were significantly enriched for *Bifidobacterium* in a group of individuals who consumed a dietary pattern containing high amounts of carbohydrates from fried white potatoes and low HEI scores. However, the “potato” consumption in the current analysis that is predictive of fecal butyrate included many forms of cooked potatoes (Figure 4A and B). It’s possible that potatoes are an underrated source of resistant starch in American diets.

We found SCFAs to be associated with markers of subclinical GI inflammation, even in healthy adults. As hypothesized, fecal SCFAs were inversely related to plasma LBP, consistent with a recent report of a smaller cohort of healthy adults in a different region of the United States [60]. The most significant relationship was an inverse association of plasma propionate and MPO, which is a marker of neutrophil infiltration in the colon. Propionate enhanced colonic regulatory T cells (Tregs) in mice to prevent colitis [61]. Therefore, we speculate that plasma propionate is a marker of higher exposure of Tregs to propionate, stimulating those Tregs to inhibit neutrophil infiltration in the colon.

The diversity, composition, and individual features of the microbiome were particularly predictive of SCFA abundance and ratios. Mirroring our findings that SCFA abundance was negatively associated with microbiome alpha diversity, healthy controls participating in a study investigating Parkinson’s disease exhibited gut microbiome diversity that was negatively correlated with SCFA abundance [62]. Furthermore, many studies investigating the impact of fiber on the microbiome find no change in alpha diversity or even a decreasing trend [[63], [64], [65], [66]]. Interestingly, we found the average variation in community composition explained by SCFA abundance to be 1.4% (Supplemental Figure 6B), similar to the average microbiome variation explained by fiber interventions in a meta-analysis of 12 studies (1.5%) [67].

The most accurate machine learning models utilized microbial taxa to predict SCFA abundance and composition. Although one study reported a low *R*^*2*^ for predicting SCFA abundance based on microbial taxonomy, with the highest *R*^*2*^ (0.14) achieved for predicting butyrate using genus data [68], our models constructed using TaxaHFE-engineered taxonomy yielded higher *R*^*2*^ values in several cases. The most successful model predicted fecal propionate with a mean *R*^*2*^ of 0.31 (Supplemental Table 3). Furthermore, we found that many of the features driving these models were known SCFA producers. For example, *Roseburia* and *Lachnospiraceae* (containing *E*. *rectale*) appeared to be highly predictive of butyrate-ratio in our cohort (Figure 5A and B) and have previously been established as important butyrate producers [69,70]. Species of *Roseburia* and *E. rectale* have been shown to prefer a lower pH environment (5.5), and increases in pH tend to favor *Bacteroides* and shift to greater propionate and acetate production [69]. Indeed, we found the entire phylum *Bacteroidetes* and class *Negativicutes*, another known propionate producer [71], to be among the top features driving ML or PERMANOVA models explaining propionate-ratio (Supplemental Figure 7). Although these observations align with our findings, it is worth noting that not all butyrate producers prefer a low pH. For example, in vitro experiments have shown *Anaerostipes caccae* to prefer higher pH (≥5.9) in order to efficiently convert lactate to butyrate [72]; however, butyrate production by *A. caccae* may also be a function of resource competition [73]. Indeed, the ecology of SCFA production is a complex cross-feeding web of primary degraders, fermenters, and even potential pH buffering taxa [71,73]. From a modeling perspective, Skwara et al. [74] showed that as the community-function landscape increases in complexity, such as complex cross-feeding networks, model predictiveness suffers. Thus, reductionist experiments designed to tease apart these ecologic parameters are critically necessary to inform future modeling efforts.

Although we expected to find that increased reads mapping to known butyrate-producing pathways to be predictive of fecal butyrate, our ML models suggested otherwise. We found no correlation between fecal butyrate or butyrate-ratio and the abundance of the acetyl-CoA fermentation to butanoate pathway or the pyruvate fermentation to butanoate pathway (Supplemental Figure 11). Others have noted a lack of correlation between SCFA functional pathways and the abundance of the metabolites themselves [75]. Pathways that were predictive of butyrate-ratio included the stachyose and inositol degradation pathways (Figure 5C; Supplemental Table 4). Foods high in stachyose include high-fiber staples such as soybeans and chickpeas [76], and inositol-containing foods include starchy vegetables, fresh fruit, and stone-ground wheat bread [77]. In a high-fiber dietary intervention, one of the only annotated pathways that increased in abundance was the inositol degradation pathway [66]. Thus, the ability to utilize particular carbon sources may be more important to butyrate synthesis than the more common butyrate synthesis genes.

Even more predictive of the butyrate-ratio was the thiamine diphosphate biosynthesis pathway iii (eukaryotes). Although we cannot explain the naming convention of this pathway, we do note that HUMAnN3 assesses the presence of this pathway using genes that also appear in bacteria - such as EC 2.7.4.7, EC 2.5.1.3, EC 2.7.6.2, and EC 3.1.3.-. Furthermore, thiamine diphosphate is a critical cofactor for the production of butyrate [78]. Our results show that the taxa containing this pathway, which also change most in abundance between high and low butyrate-ratio individuals, include *R. faecis* and *Roseburia intestinalis* (Figure 5D). Butyrate-producer *R. faecis,* in particular, has been shown to be prototrophic for thiamine, whereas many other butyrate-producing microbes are auxotrophic [79]. It is worth noting that although our results show *R. intestinalis* contributing to the thiamine diphosphate pathway abundance, this species appears to be auxotrophic for thiamine despite containing all the genes in the pathway [79]. Because the pathway for thiamine diphosphate biosynthesis was so predictive of butyrate-ratio, we then wondered if dietary thiamine (from food or supplements) would also be predictive of butyrate. However, we found no correlation between dietary thiamine and fecal butyrate-ratio (Supplemental Figure 12). Thiamine is necessary for many human enzymes involved in metabolism. As such, it is readily absorbed in the small intestine, leaving little for the gut microbiota residing in the colon [80,81]. However, thiamine-producing microbes appear to produce enough thiamine as a “public good” to sustain neighboring auxotrophs [82]. Our data suggests that the combination of butyrate producers *and* thiamine prototrophs results in a higher fecal butyrate-ratio.

Our study has a few notable caveats. First, as an observational study, ours is not capable of identifying causal relationships between the biomarkers measured and SCFA abundance or composition. We also acknowledge that these data represent a “snapshot” of incredibly dynamic processes. To better test the hypotheses our study generates, dietary interventions coupled with longitudinal sampling are warranted. Furthermore, although we identified significant correlations between SCFAs and diet or biomarkers, the small degree of these correlations indicates that further research is needed to establish meaningful relationships between diet and the physiologic metrics influenced by diet. Finally, although machine learning algorithms substantially aid our ability to understand complex data, these algorithms are not without pitfalls [83]. For one, ML models perform best with a large number of samples – a requirement not easily met for many human studies, including our own. We utilized feature engineering to reduce the dimensionality of our data, especially the redundancy of hierarchically organized features. Applying hierarchical feature engineering prior to downstream machine learning allows us to best understand patterns in our data but biases the model and reduces its generalizability due to “data leakage.” As mentioned in our methods, we apply TaxaHFE to all the data and also solely to the training data (reduced bias) and present both model scores in an effort to be more transparent.

Overall, our findings indicate that SCFA production is dependent on the availability of substrates from specific foods together with microbes that can use those substrates and synthesize or obtain the necessary enzymatic cofactors. These results illustrate the complex biology underpinning SCFA production in the human gut.

## Acknowledgments

We thank Eduardo Cervantes, Ellen Bonnel, and the USDA Nutritional Phenotyping team for assisting in data collection, and the USDA Bioanalytical Support Laboratory for sample management and stool processing. We also thank the Biomarkers Core of the Irving Institute for Clinical and Translational Research of Columbia University Medical Center for measurements of plasma short-chain fatty acids.

## Author contributions

The authors’ responsibilities were as follows – DGL: conceptualization; AO, ZA, CBS, JWN, MEK, DGL: methodology; AO: software; ZA: validation; AO: formal analysis; ZA, CBS, JWN, MEK, DGL: investigation; JWN: resources; ZA: data curation; AO, DGL: writing - original draft; ZA, CBS, JWN, MEK: writing - review and editing; AO: visualization; DGL, CBS: supervision; DGL, CBS: project administration; and all authors: read and approved the final manuscript.

## Conflict of interest

CBS is an editorial board member of The Journal of Nutrition and played no role in the Journal’s evaluation of the manuscript. All other authors report no conflicts of interest.

## Funding

This research was primarily supported by USDA Agricultural Research Service (ARS) grant 2032-51530-026-00D and 2032-10700-002-00D.

AO was supported by appointment to the research participation program at the ARS, USDA, administered by the Oak Ridge Institute for Science and Education (ORISE) through an interagency agreement between the United States Department of Energy (DOE) and ARS. ORISE is managed by Oak Ridge Associated Universities (ORAU) under DOE contract number DE-SC0014664. All opinions expressed in this paper are the author’s and do not necessarily reflect the policies and views of USDA, DOE, or ORAU/ORISE. The metagenomic library preparation and sequencing were carried out at the DNA Technologies and Expression Analysis Cores at the University of California Davis Genome Center, supported by NIH shared instrumentation grant 1S10OD010786-01. This research also used resources provided by the SCINet project of the USDA ARS, ARS project number 0500-00093-001-00-D. USDA ARS is an equal-opportunity employer.

## Data availability

Metagenomic reads for 330 individuals are deposited in the NCBI Sequence Read Archive under two accession numbers: SRP354271 and SRP497208. Requests for nonmetagenomic data from the USDA Agricultural Research Service Western Human Nutrition Research Center (WHNRC) Nutritional Phenotyping Study used in this analysis should be made via an email to the senior WHNRC author on the publication of interest. Requests will be reviewed quarterly by a committee consisting of the study investigators. Code to reproduce our results can be found in a GitHub repository: https://github.com/aoliver44/SCFA-Analysis. A docker container containing the R packages used for data wrangling, statistical analyses, and visualizations is also provided to aid in reproducibility.
