# Supplementary material for: Diet, Microbiome, and Inflammation Predictors of Fecal and Plasma Short-Chain Fatty Acids in Humans
Source: J Nutr. 2024 Aug 20;154(11):3298–311. doi: 10.1016/j.tjnut.2024.08.012 (PMC11600052; doi:10.1016/j.tjnut.2024.08.012)
Supplement: multimedia component 2 [file mmc2.pdf]

Online supporting material
Title: Diet, microbiome, and inflammation predictors of fecal and plasma short-chain fatty acids in humans

Authors: Andrew Oliver[1], Zeynep Alkan[1], Charles B. Stephensen[1,2], John W. Newman[1,2,3], Mary E. Kable[1,2], Danielle G. Lemay [1,2,3]*

Supplemental Table Legends
Supplemental Table 1: Age, sex, and BMI sample numbers for fecal and plasma SCFA samples analyzed.

Supplemental Table 2: Partial correlation results between SCFAs and dietary variables related to healthy eating and fiber abundance and diversity. For plasma butyrate and propionate, tobit models were used for censored regression. Covariates for plasma SCFA models were age, sex, BMI. For fecal SCFA models, covariates were age, sex, BMI, stool weight and stool consistency (Bristol stool score). Regression p-values were family-wise adjusted within each SCFA and whether inflammation samples were kept or dropped, using the false discovery method.

Supplemental Table 3: Raw ML model scores.

Supplemental Table 4: Absolute SHAP values for the top 10 most predictive features in the models whose seed resulted in the best (lowest MAE score). The column “correlation direction” indicates “+” if the variable and the SCFA directly varied and “-” if the variable and the SCFA inversely varied.

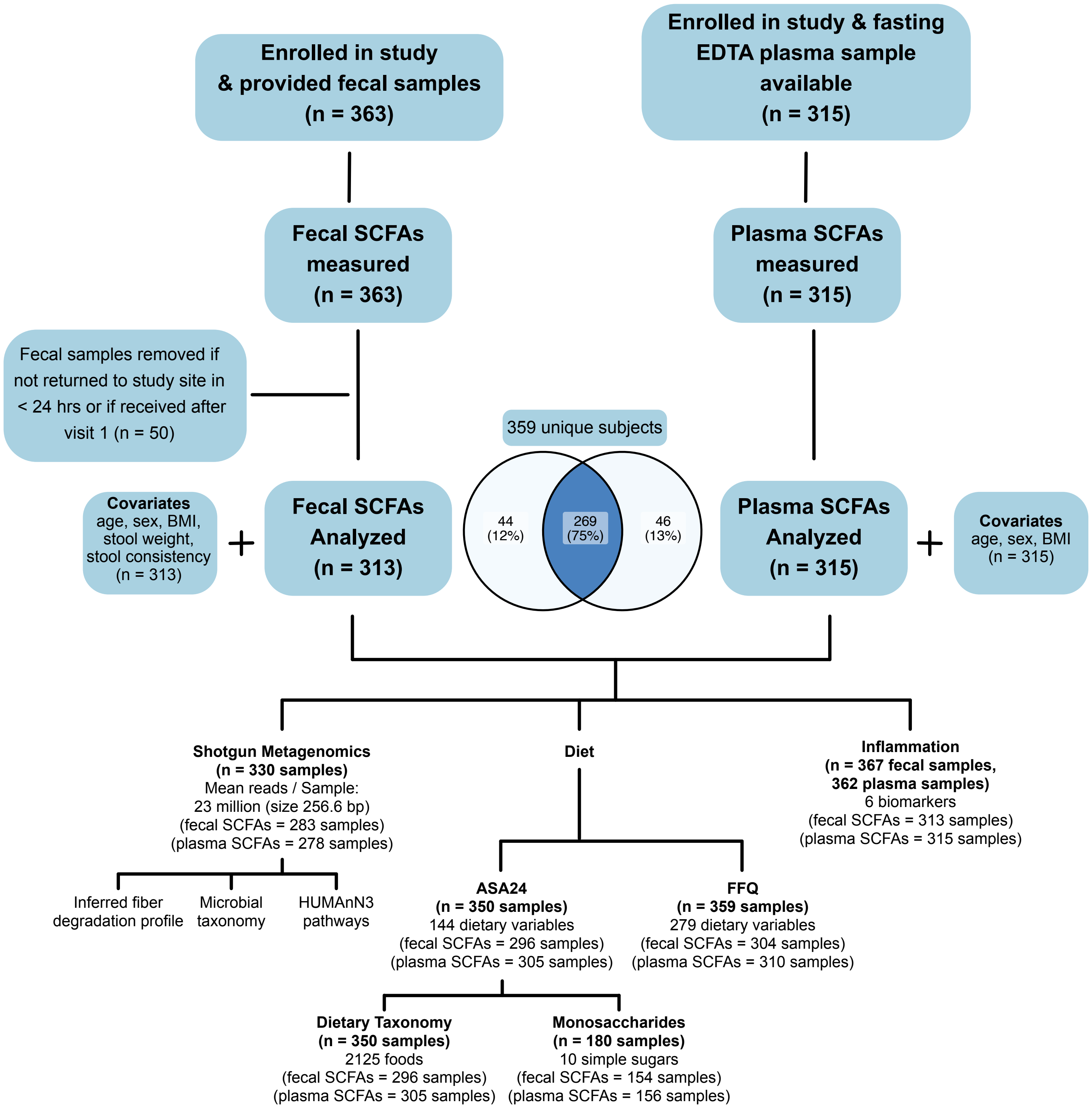

Supplemental Figure 1: STROBE diagram illustrating number of samples and variables analyzed across the different datasets.

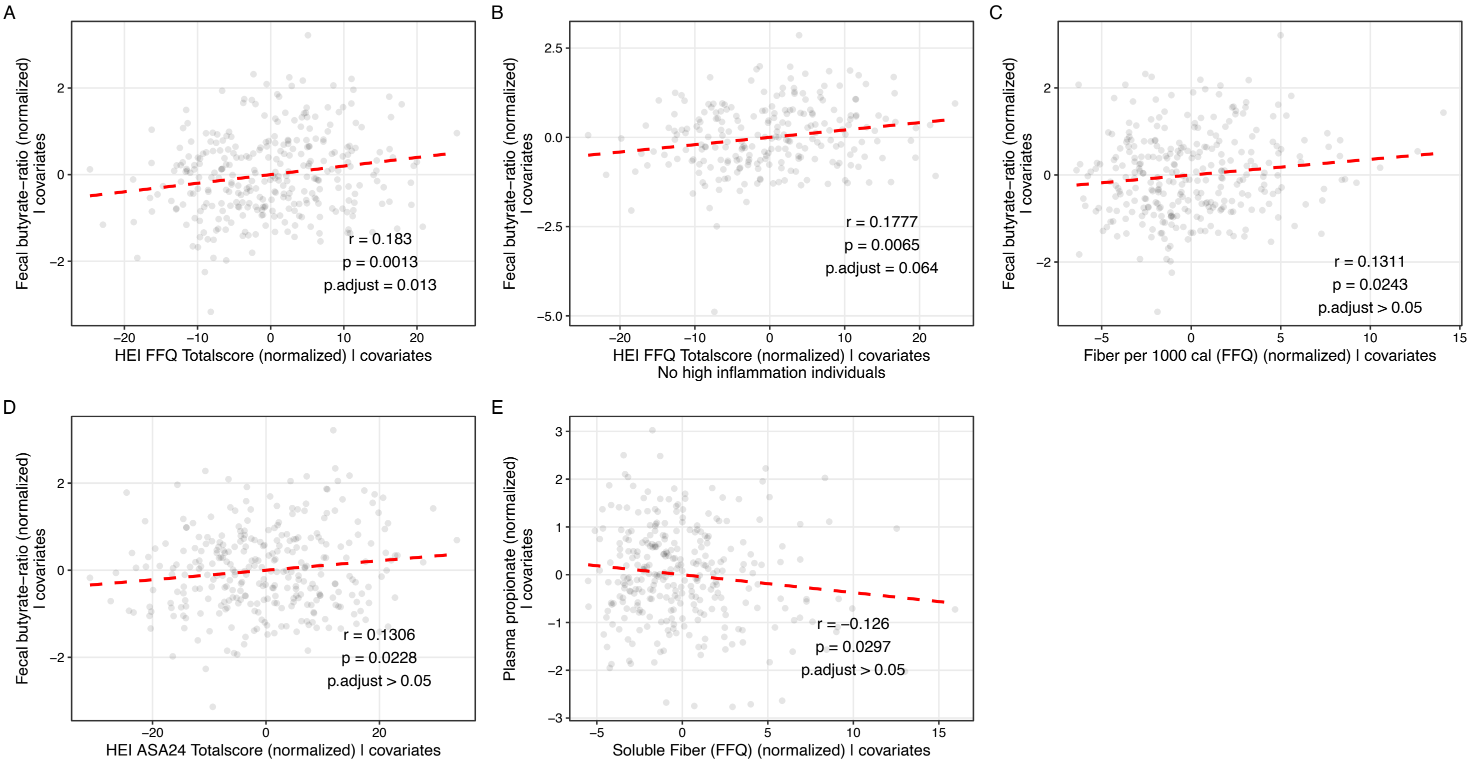

Supplemental Figure 2: Partial correlation between HEI total score (FFQ) and fecal butyrate-ratio with all individuals **(A)** or without individuals with frank inflammation **(B)**. **C)** Partial correlation between calorie corrected fiber intake (FFQ) with fecal butyrate-ratio. **D)** Partial correlation between HEI total score (ASA24) with fecal butyrate-ratio. **E)** Partial correlation between total soluble fiber intake (FFQ) with plasma propionate. Partial correlations models contained fecal covariates (age, sex, BMI, stool weight, and stool consistency) or a subset (age, sex, BMI) for plasma covariates.

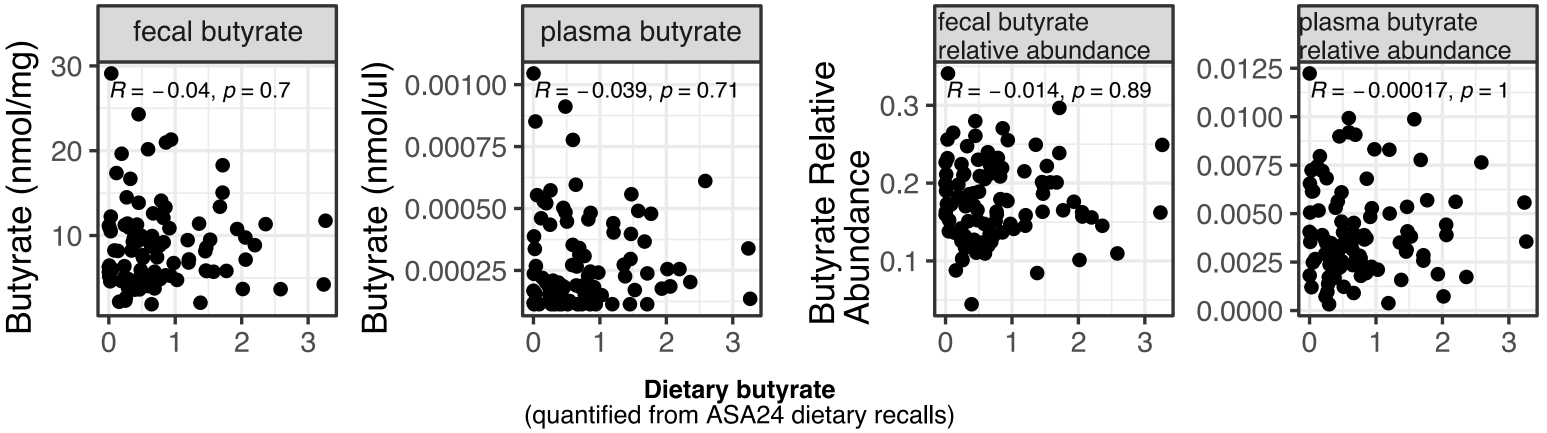

Supplemental Figure 3: Correlation between butyrate quantified from the ASA24 recalls and either fecal or plasma butyrate or butyrate relative abundance.

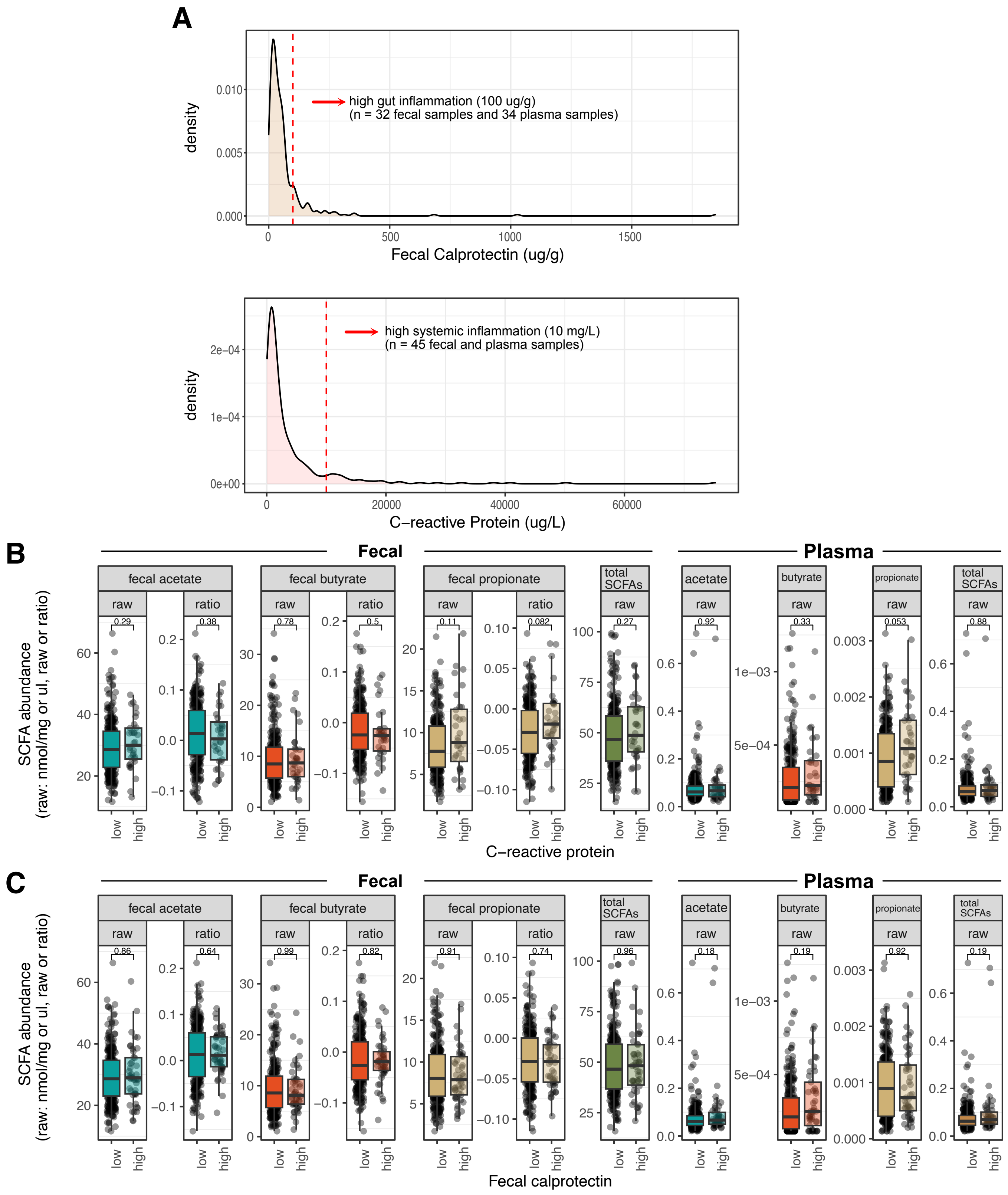

Supplemental Figure 4: A) Distribution of individuals and their measures of fecal calprotectin (top) or plasma C-reactive protein (bottom). **B)** Differences in SCFA abundance or composition between individuals with normal or high levels of C-reactive protein. **C)** Differences in SCFA abundance or composition between individuals with normal or high levels of fecal calprotectin.

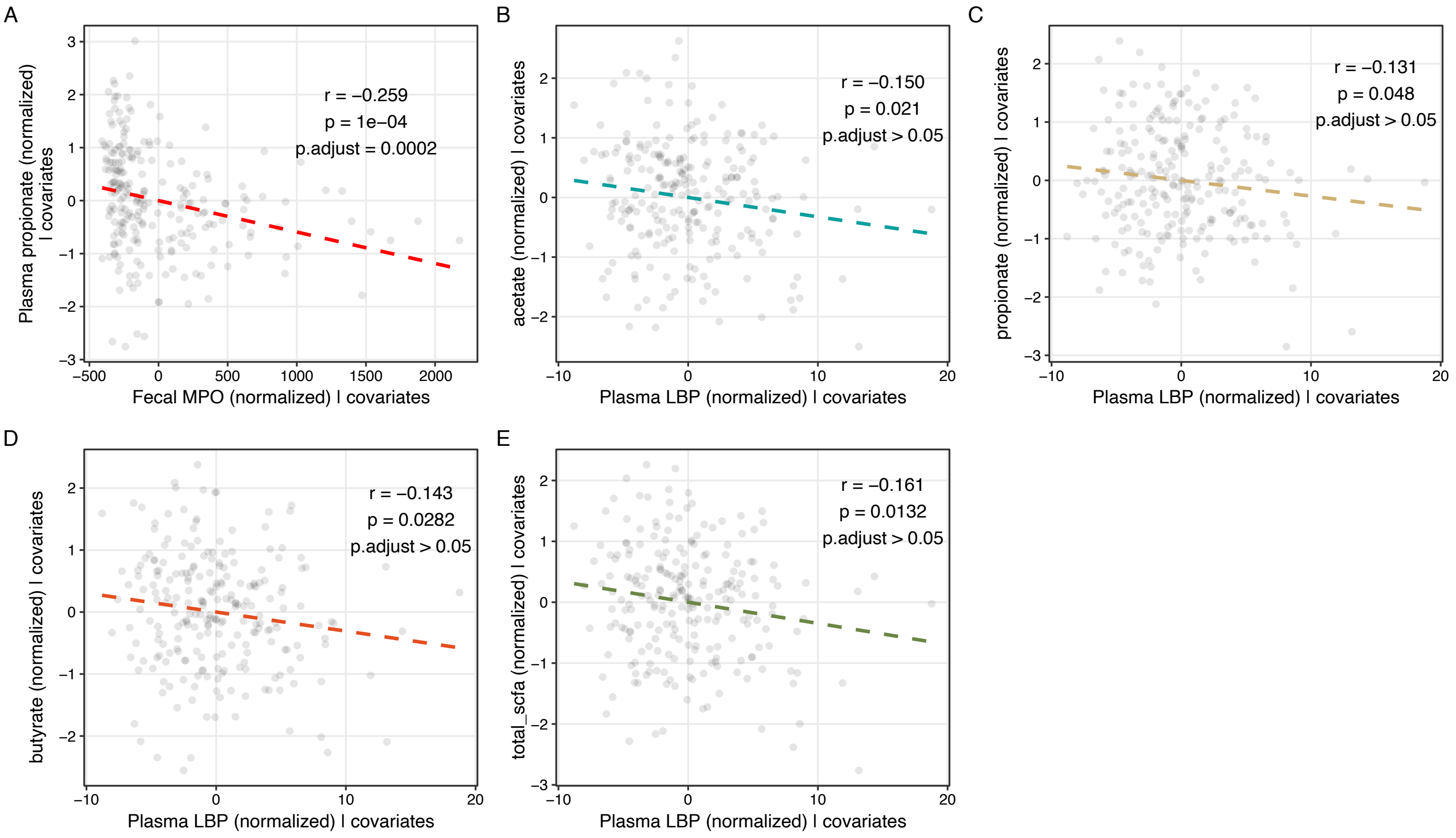

Supplemental Figure 5: A) Partial correlation between fecal MPO and plasma propionate. Partial correlation between plasma lipopolysaccharide binding protein and **B)** fecal acetate, **C)** fecal propionate, **D)** fecal butyrate, and **E)** total fecal SCFAs. Partial correlations models contained fecal SCFA covariates (age, sex, BMI, stool weight, and stool consistency) or a subset (age, sex, BMI) for plasma SCFA covariates.

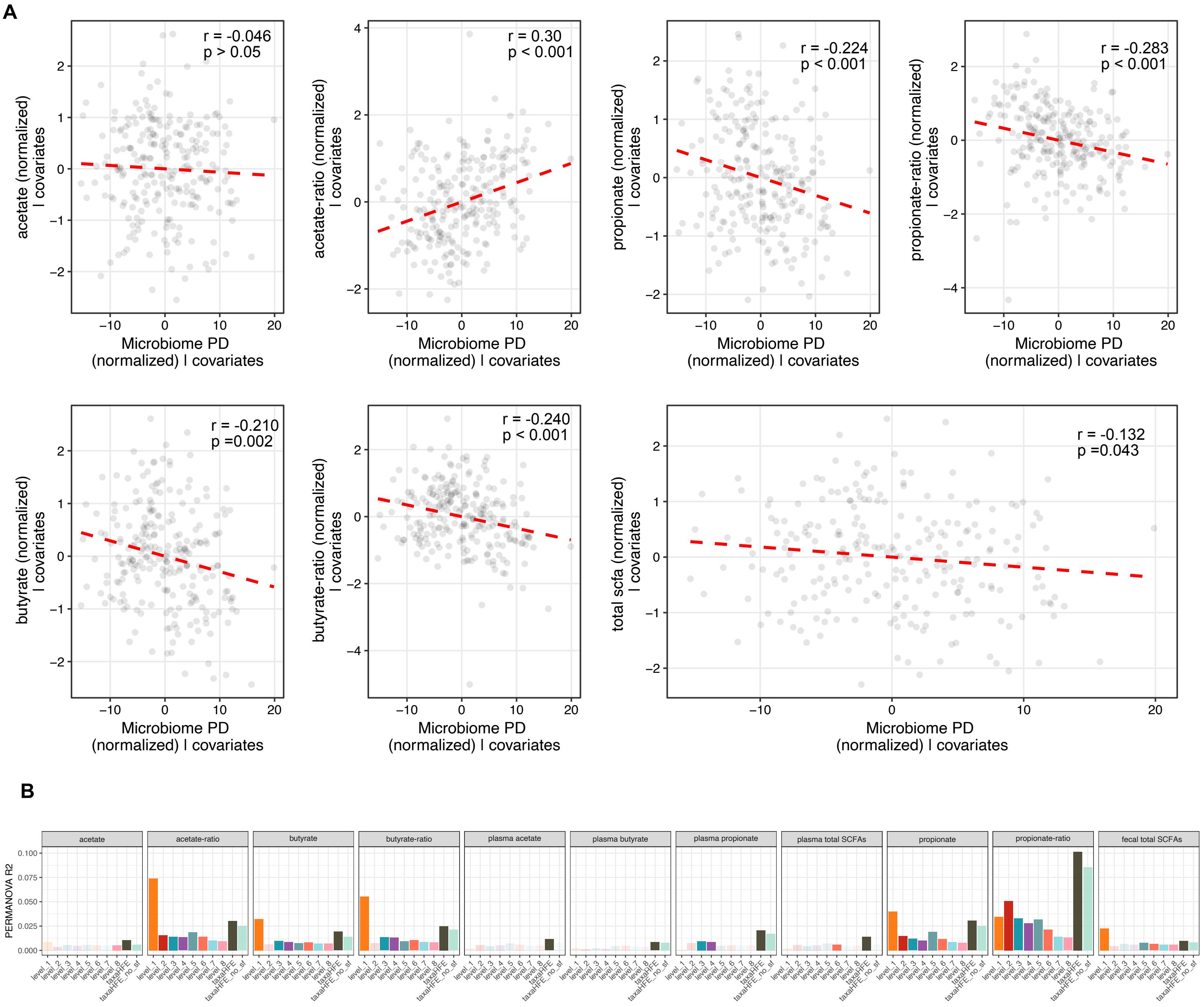

Supplemental Figure 6: A) Partial correlations between the phylogenetic diversity of microbiomes and fecal SCFAs. **B)** The R2 from PERMANOVA models assessing the variation in community composition attributed to SCFAs. Transparent bars are PERMANOVA models where the SCFA did not explain significant variation ($p > 0.05$). Each taxonomic level was run, along with TaxaHFE-engineered microbiome taxonomy. Partial correlations models contained fecal SCFA covariates (age, sex, BMI, stool weight, and stool consistency).

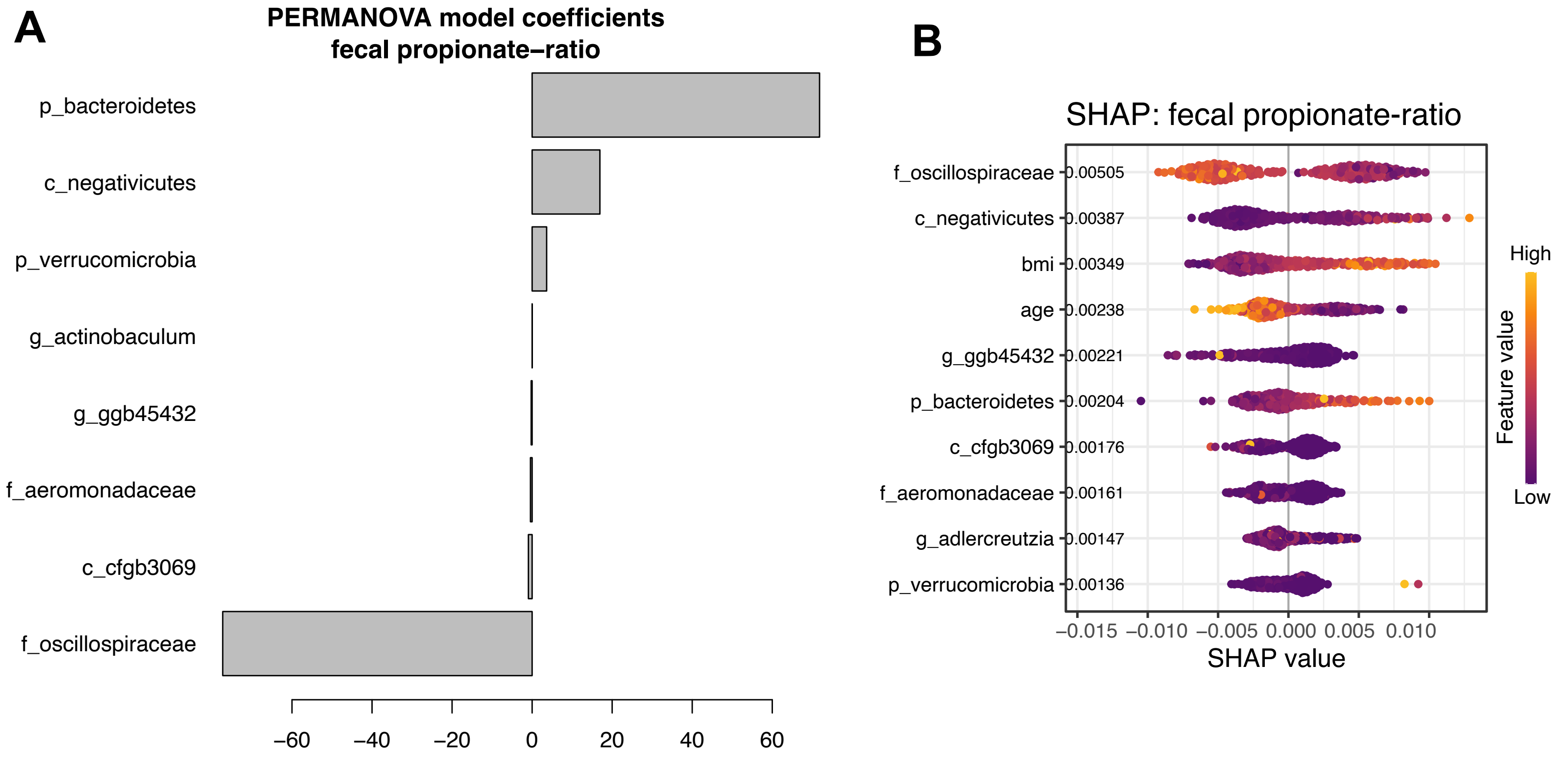

Supplemental Figure 7: A) PERMANOVA coefficients for the model assessing the variability in TaxaHFE-engineered microbial community composition with respects to fecal propionate-ratio. **B)** A Shapley beeswarm plot showing the most predictive features in a machine learning module using TaxaHFE-engineered microbiome taxonomy to predict fecal propionate-ratio.

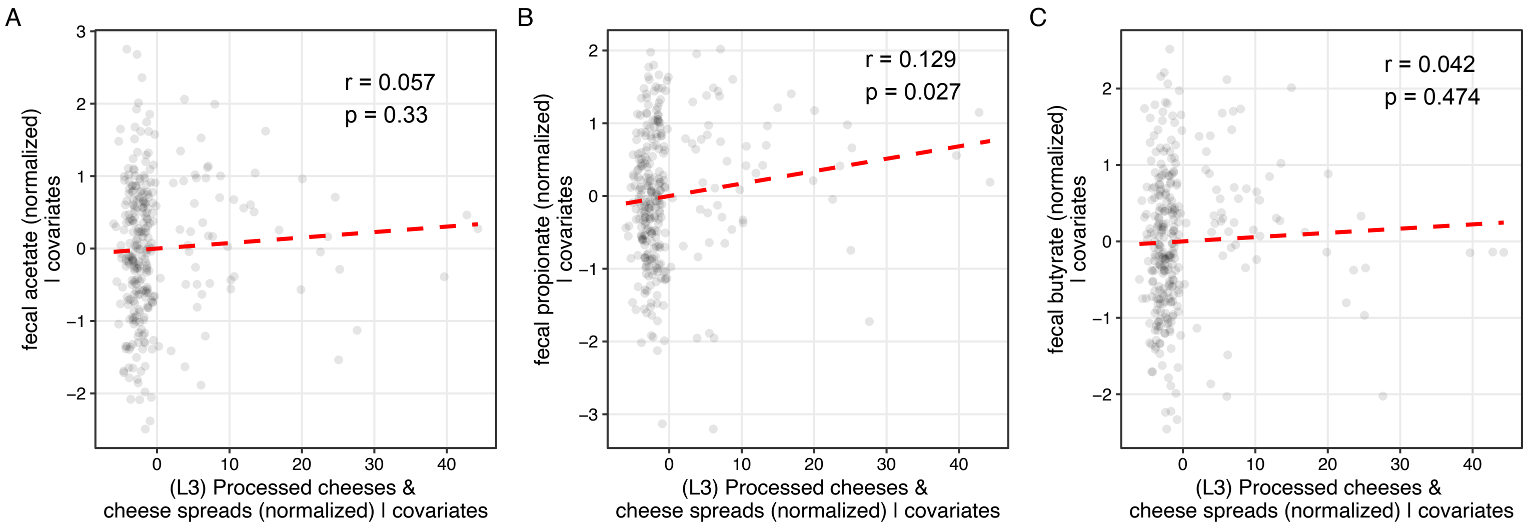

Supplemental Figure 8: The partial correlations between L3 processed cheeses and cheese spreads with **A)** fecal acetate, **B)** fecal propionate, and **C)** fecal butyrate. Partial correlations models contained fecal SCFA covariates (age, sex, BMI, stool weight, and stool consistency).

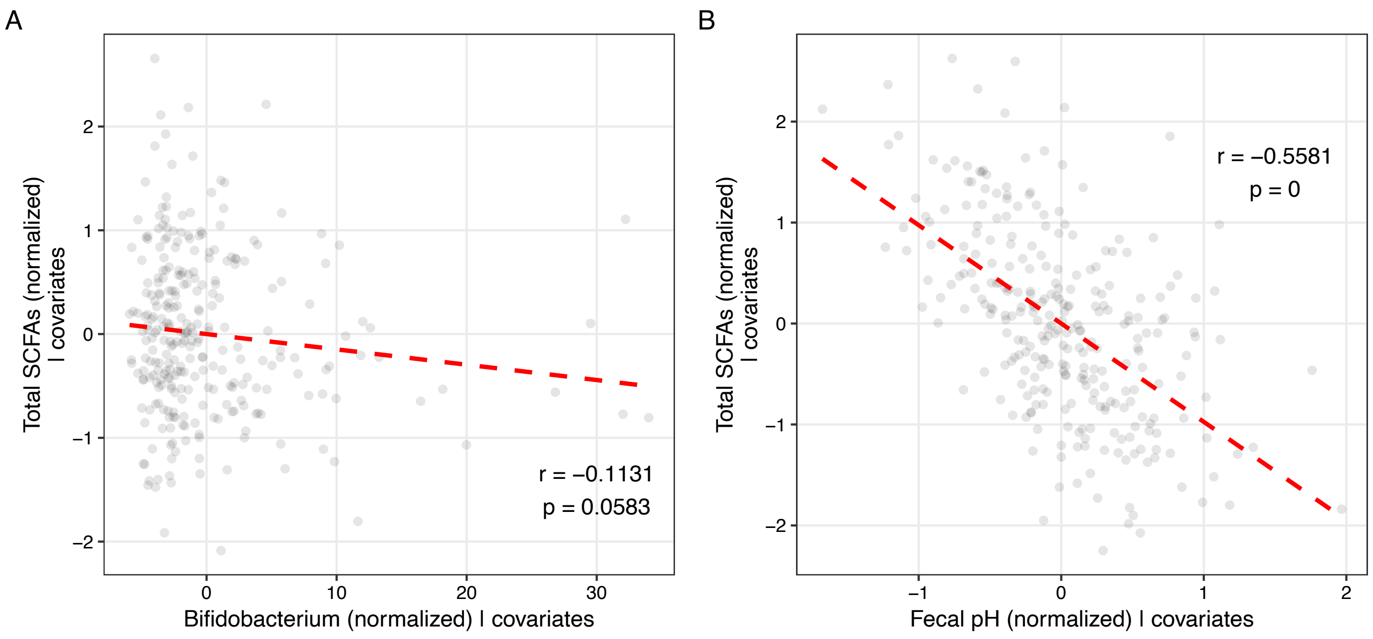

Supplemental Figure 9: A partial correlation model with fecal SCFA covariates (age, sex, BMI, stool weight, and stool consistency) and both *Bifidobacterium* relative abundance and fecal pH. The partial correlation between *Bifidobacterium* **(A)** and fecal pH **(B)** with total SCFAs is plotted.

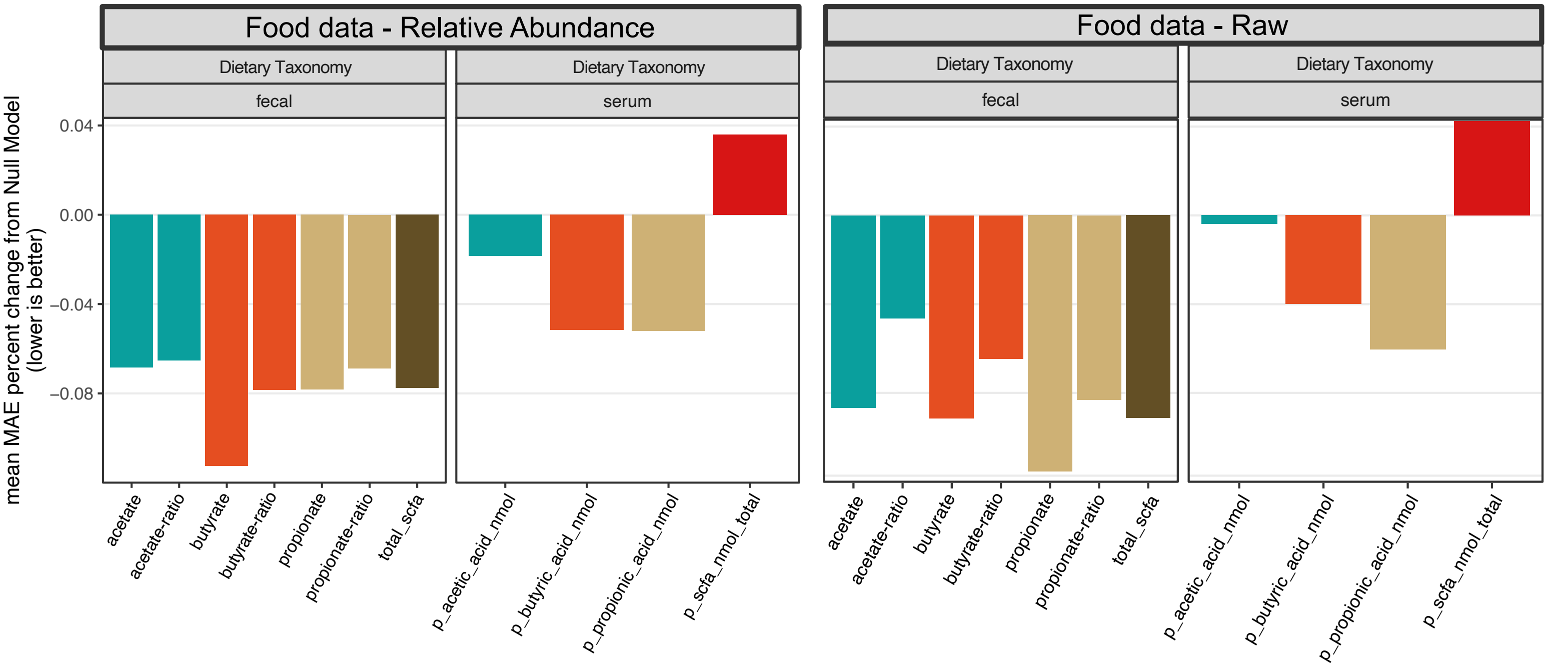

Supplemental Figure 10: The difference in mean MAE percent change from null models when predicting SCFAs from food data that has been total sum scaled (left) or not scaled (right). Note that in both cases, raw fecal SCFAs (and not ratios) have better (lower) scores.

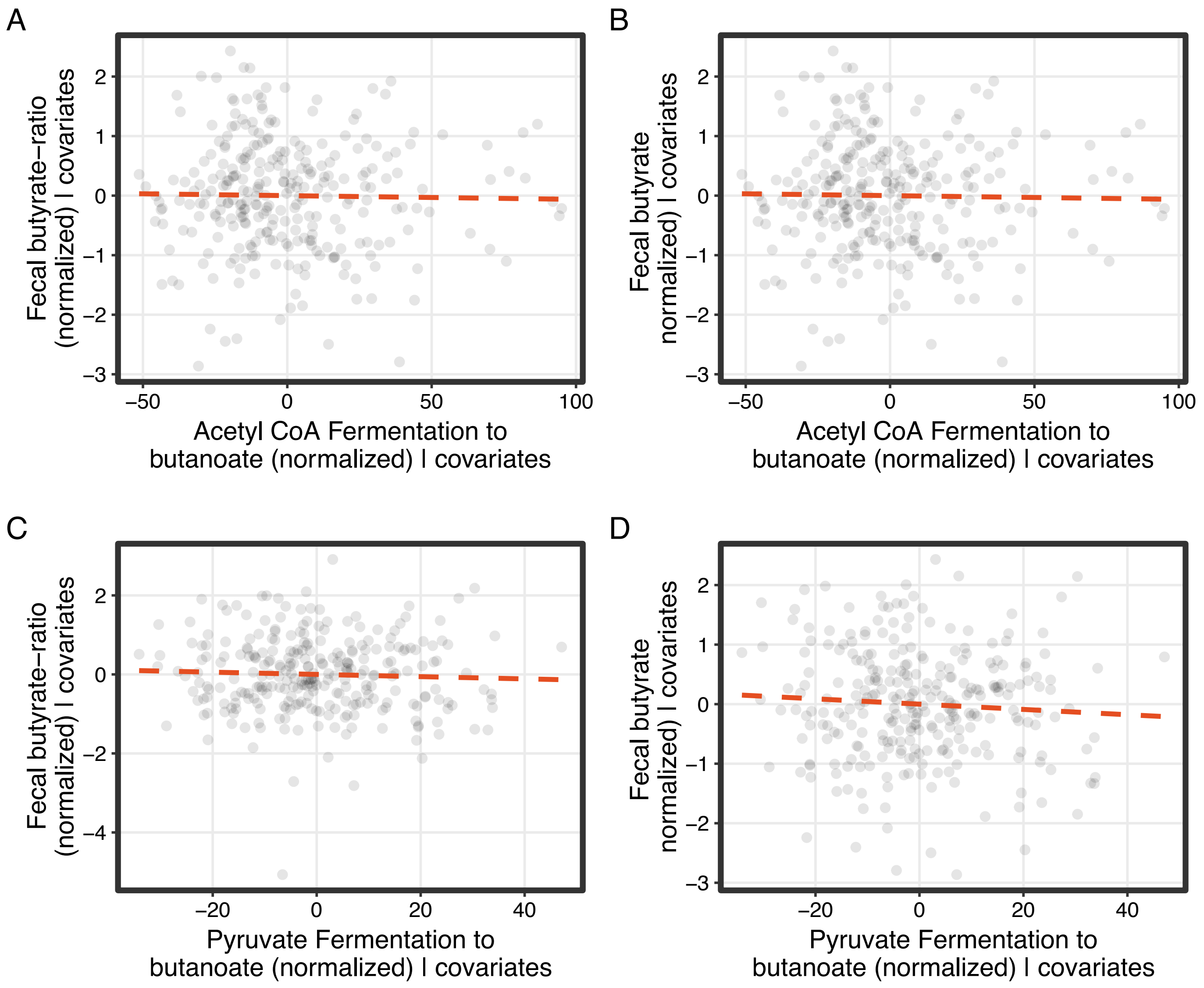

Supplemental Figure 11: The partial correlation between the pathway acetyl CoA fermentation to butanoate with **A)** fecal butyrate-ratio and **B)** fecal butyrate. Also shown is the partial correlation between the pathway pyruvate fermentation to butanoate with **C)** fecal butyrate-ratio and **D)** fecal butyrate. Partial correlations models contained fecal SCFA covariates (age, sex, BMI, stool weight, and stool consistency).

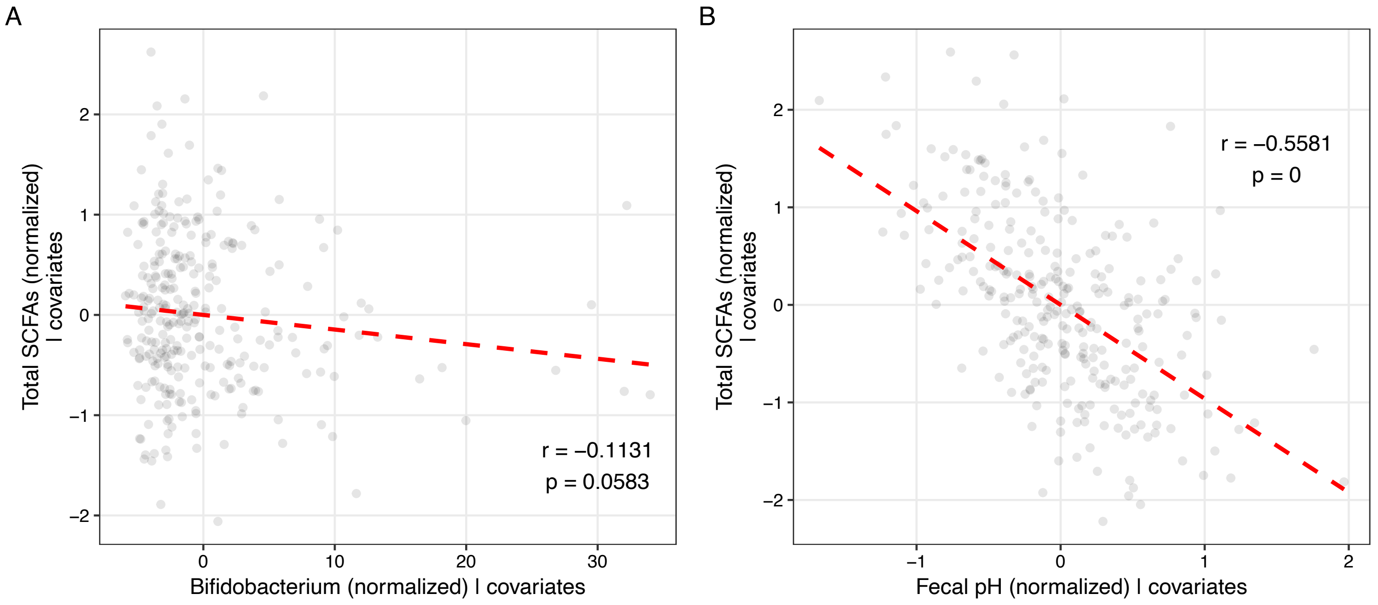

Supplemental Figure 12: The partial correlation between fecal butyrate-ratio and the **A)** thiamine diphosphate biosynthesis pathway and **B)** dietary thiamine (from foods and supplements). Partial correlations models contained fecal SCFA covariates (age, sex, BMI, stool weight, and stool consistency).
